# Drug Repositioning for Diabetes Based on 'Omics' Data Mining

**DOI:** 10.1371/journal.pone.0126082

**Published:** 2015-05-06

**Authors:** Ming Zhang, Heng Luo, Zhengrui Xi, Ekaterina Rogaeva

**Affiliations:** 1 Department of Medicine, Division of Neurology, Tanz Centre for Research in Neurodegenerative Diseases, University of Toronto, 60 Leonard Street, Toronto, Ontario, M5T 2S8, Canada; 2 University of Arkansas at Little Rock/University of Arkansas for Medical Sciences Bioinformatics Graduate Program, 2801 S. University Ave., Little Rock, AR, 72204, United States of America; Stanford University School of Medicine, UNITED STATES

## Abstract

Drug repositioning has shorter developmental time, lower cost and less safety risk than traditional drug development process. The current study aims to repurpose marketed drugs and clinical candidates for new indications in diabetes treatment by mining clinical ‘omics’ data. We analyzed data from genome wide association studies (GWAS), proteomics and metabolomics studies and revealed a total of 992 proteins as potential anti-diabetic targets in human. Information on the drugs that target these 992 proteins was retrieved from the Therapeutic Target Database (TTD) and 108 of these proteins are drug targets with drug projects information. Research and preclinical drug targets were excluded and 35 of the 108 proteins were selected as druggable proteins. Among them, five proteins were known targets for treating diabetes. Based on the pathogenesis knowledge gathered from the OMIM and PubMed databases, 12 protein targets of 58 drugs were found to have a new indication for treating diabetes. CMap (connectivity map) was used to compare the gene expression patterns of cells treated by these 58 drugs and that of cells treated by known anti-diabetic drugs or diabetes risk causing compounds. As a result, 9 drugs were found to have the potential to treat diabetes. Among the 9 drugs, 4 drugs (diflunisal, nabumetone, niflumic acid and valdecoxib) targeting COX2 (prostaglandin G/H synthase 2) were repurposed for treating type 1 diabetes, and 2 drugs (phenoxybenzamine and idazoxan) targeting ADRA2A (Alpha-2A adrenergic receptor) had a new indication for treating type 2 diabetes. These findings indicated that ‘omics’ data mining based drug repositioning is a potentially powerful tool to discover novel anti-diabetic indications from marketed drugs and clinical candidates. Furthermore, the results of our study could be related to other disorders, such as Alzheimer’s disease.

## Introduction

Diabetes mellitus is one of the most prevalent diseases in the world, affecting approximately 382 million people around the world in 2013, costing at least $548 billion in 2013 according to the international diabetes federation (IDF). Diabetic drug safety is a big concern during the development of new drugs. Avandia from GSK, for example, was found to be associated with risk of heart attack [1], resulting in a recommendation of suspension by European Medicines Agency (EMA) in 2010. Aleglitazar from Roche, a Peroxisome proliferator-activated receptor gamma (PPARG) agonist, was terminated in phase III clinical trial in 2013 due to safety concerns for bone fractures, heart failure and gastrointestinal bleeding. Among the current diabetic drug developmental pipelines in leading pharmaceutical companies, 24 drugs have survived the early stages of drug development (phase I, II clinical trials) and are now in phase III clinical trials or post-market surveillance. Among the 24 drugs, 17 (71%) are incretin analogs, DPP4-inhibitors or insulin analogs (S1 Table). However, the association between incretin therapy and risk of pancreatitis and cancer is still uncertain and under investigations by the FDA and EMA [2]. It has been long recognized that the traditional drug development process requires a lot of time (10–17 years) and is extremely costly, but has a low success rate (< 10%) and high safety risk. Therefore, novel strategies are needed for developing novel diabetic drugs in a more efficient way with lower safety risks.

Drug repositioning (or repurposing) has long been used in the drug development process by reusing marketed drugs and clinical candidates for a new indication (such as treating another disease) [3]. Compared to *de novo* drug discoveries, drug repositioning may tremendously reduce the development time to 3–12 years, cost and safety risks. For instance, most repositioned candidates have already been assessed by phase I or II clinical trials regarding their original indications [4]. Therefore, toxicity information in animals and humans is often available.

There are multiple approaches for drug repositioning. The “Disease Focus” approach, for example, employs experimental data related to diseases (e.g. ‘omics’ data) and knowledge of how drugs modulate phenotypes related to diseases (e.g. side effects). Several methods, such as expression pattern comparison [5] (connectivity map, CMap), text mining [6] and networks analysis [7], have been established for mining ‘omics’ data. Meanwhile, computational methods have been applied to predict drug-protein interactions [8], drug off-targets [9] and drug side effects [10]. Recently, scientists started to use data from genome wide association studies (GWAS) [11] and pathogenesis knowledge from the Online Mendelian Inheritance in Man (OMIM) database [12] to perform drug repositioning.

With the technological advancement in genomics, proteomics and metabolomics, biomedical data are quickly emerging and can be utilized as a valuable resource for drug repositioning. GWAS data has been successfully utilized for drug repositioning [11]. Proteomics, assessing the whole proteome in cells, tissues or body fluids, is involved in different stages of target-based and phenotype-based drug discoveries, including target selection, target validation, lead selection/optimization and preclinical testing. Metabolomics plays an important role in translational medicine, preclinical research/biomarker discovery, and patient stratification [13]. Proteins are the most common targets of small compound drugs. Therefore, data from metabolomics and proteomics studies is a valuable resource for drug repositioning. However, no such effort has been made so far. The current study aims to systematically integrate GWAS, proteomics and metabolomics data for drug repositioning in diabetes treatment.

## Materials and Methods

### 2.1 Literature search and data extraction

To obtain information on diabetes related genes, proteins and metabolites, we searched the PubMed database up to August 2014 using the keywords “diabetes and GWAS”, “diabetes and proteomics”, “diabetes and protein”, “diabetes and metabolomics”, “diabetes and metabolites”. We included the literature in our study according to the following criteria: 1) all samples have to be human samples, such as serum, plasma or tissues; 2) the clinical phenotype has to be “type 1 diabetes” or “insulin dependent diabetes mellitus”, “type 2 diabetes”, “gestational diabetes”, “impaired glucose tolerance”, “impaired fasting glycemia” or “insulin resistance”.

For the GWAS studies, we extracted information on 1) the genes associated with diabetes; 2) the SNPs; 3) patient ethnicity; 4) the phenotypes (diabetes type). For the proteomics studies, we extracted the following information: 1) the proteins associated with diabetes; 2) the direction of the change in protein level; 3) the methods used for measuring protein level; 4) the sample types; 5) the phenotypes. For the metabolomics studies, we extracted information on 1) the metabolites associated with diabetes; 2) the direction of the change in metabolite levels, 3) the sample types, 4) patient ethnicity, 5) the methods used for assessing the metabolites and 6) the phenotypes.

### 2.2 Mining diabetic metabolites related proteins

*In vivo*, enzymes and transporters are two groups of proteins directly associated with the turnover of human metabolites. By searching the Human Metabolome Database (HMDB, http://www.hmdb.ca), we obtained the names of enzymes or transporters associated with diabetes related metabolites that were discovered from previous metabolomics studies.

### 2.3 Constructing the diabetic metabolites-proteins network

To visualize the association between diabetic metabolites and their corresponding enzymes or transporters, Cytoscape was used (www.cytoscape.org) to construct the metabolites-proteins network [14].

### 2.4 Mapping diabetes risk proteins to proteins with drug projects

Diabetes related genes or proteins retrieved from genomics and proteomics studies were combined with proteins related to diabetic metabolites retrieved from metabolomics data to generate a set of diabetic risk proteins. The Therapeutic Target Database (TTD version 4.3.02) contains information on 236 targets of 20667 drugs at the stages of approved, clinical trial and experimental. TTD was used to assess if those diabetes risk proteins have drug projects available [12]. Therefore, we selected diabetic risk proteins with drug projects to gather information on the 1) drug target, 2) current disease indication, 3) drug name, 4) drug development stage and 5) drug action mode. To focus on those most promising drugs to be repurposed in diabetes therapy, targets/drugs that are at the research or preclinical stages were excluded. Targets/drugs at the approved stage or in clinical trials were included in the following studies.

### 2.5 Application of pathogenesis information into anti-diabetic drug repositioning

Most drugs are either antagonist or agonist, therefore the pathogenesis of target proteins is a key basis for predicting if the drug may improve or worsen the disease phenotype [12]. We employed the OMIM (http://www.omim.org) and a literature search (PubMed) to gather knowledge on the pathogenesis of the anti-diabetic targets. Specifically, the gain of function (GOF) and the loss of function (LOF) roles in human or animal models were gathered to select anti-diabetes protein targets [15–24]. To take advantage of this strategy, we excluded those drugs with evidence of aggravating diabetic symptoms. For example, if drug D activates target T, and GOF of target T was known to increase diabetes risk, then drug D is more likely to cause diabetes instead of treating it.

### 2.6 CMap analysis

The Connectivity Map (CMap) is a collection of genome-wide transcriptional expression data from cultured human cells treated with compounds, and simple pattern-matching algorithms [5]. In the current study, the candidate drugs were input into CMap to evaluate if it is positively associated with known anti-diabetic drugs (e.g. metformin) or reversely associated with known diabetes risk compounds (e.g. streptozocin).

## Results

### 3.1 Omics studies revealed diabetes related genes, proteins and metabolites

**Fig 1:**
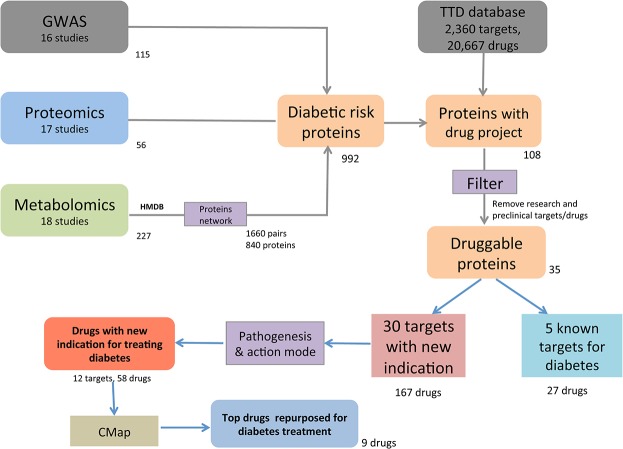
Flow-chart of drug repositioning by mining ‘omics’ data. We retrieved 17 GWAS studies, 18 proteomics studies and 19 metabolomics studies that assessed diabetes patients until August 2014. 115 genes, 56 proteins and 227 metabolites were significantly associated with diabetes. An HMDB search revealed 1660 metabolite-protein pairs corresponding to 840 proteins. Overall, 992 unique proteins associated with diabetes were gathered and mapped to the TTD database and 108 of them had drug projects information. After removing those under experimental and preclinical stages, we obtained 35 protein targets, including 5 known anti-diabetic targets (27 drugs projects) and 30 unknown anti-diabetic targets (167 drugs projects). Pathogenesis knowledge was retrieved from the OMIM and Pubmed databases, 12 targets corresponding to 58 drugs were indicated to have novel indication for diabetes treatment. CMap analysis indicated that 9 of the 58 drugs have the potential to treat diabetes.

By searching Pubmed, we included in the current study 16 GWAS papers, 17 proteomics studies and 18 metabolomics papers studying diabetes (Fig 1). We selected 115 genes, 56 proteins and 227 metabolites that were reported to be significantly associated with diabetes or impaired glucose metabolism in humans (Fig 1, S2–S4 Tables).

### 3.2 Visualization of metabolite-protein network associated with diabetes

**Fig 2:**
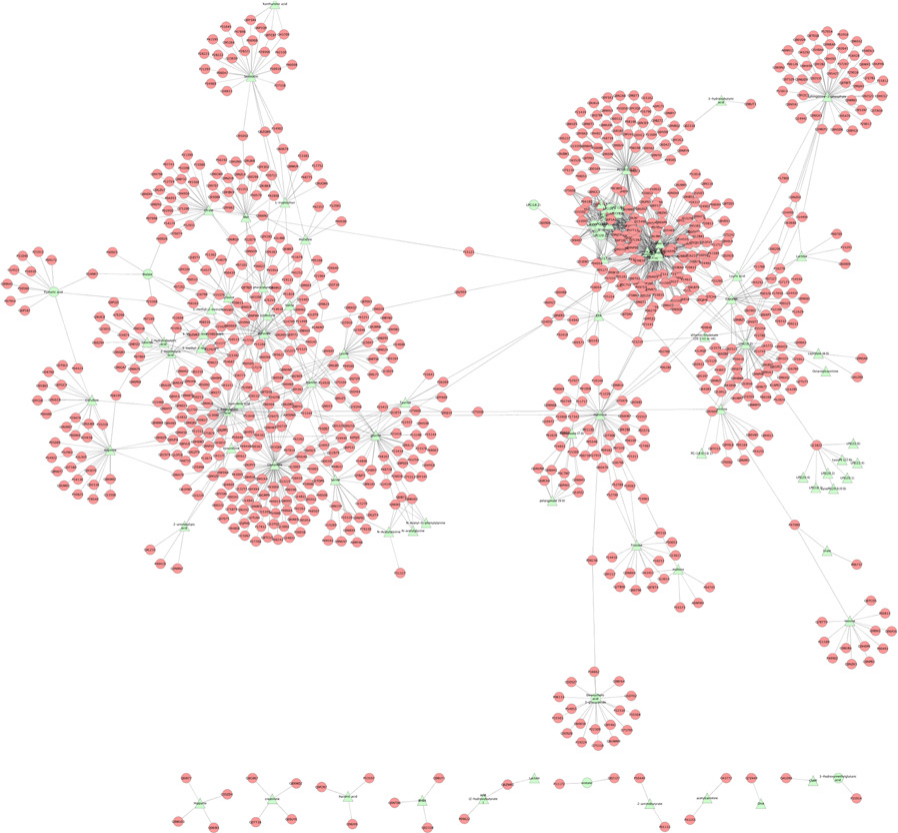
Diabetic metabolite-protein network. The Cytoscape tool was used to generate the diabetes associated metabolites and their connections to metabolic enzymes/transporters. Overall 1660 diabetes related metabolite-protein pairs were established and visualized. Green triangles represent metabolites associated with diabetes, and red circles represent proteins associated with metabolites based on HMDB database.

The 227 diabetes associated metabolites revealed from the metabolomics studies were linked to 840 enzymes or transporters (1660 metabolite-protein pairs) based on the HMDB (The Human Metabolome Database, http://www.hmdb.ca). The metabolite-protein network was generated using Cytoscape (V3.1.1) (Fig 2) and shows the highly connected metabolic pathways of various metabolites.

### 3.3 Diabetes risk proteins mapping to drug projects

840 metabolic proteins associated with diabetic metabolites were combined with 115 genes and 56 proteins, leading to 992 unique diabetic risk proteins. Uniprot IDs were retrieved to map the corresponding proteins. A TTD database search showed that 108 of the 992 proteins have at least one drug project. To focus on the most promising candidates, we filtered out those drugs projects at the research or preclinical stage, because they only had information from *in vitro* or animal model experiments and had no toxicity information in humans. Therefore, we selected 35 druggable proteins for the following studies (Fig 1).

### 3.4 Five known and thirty unknown anti-diabetic drug targets discovered using the current repositioning strategy

Five of the 35 proteins (14.3%) (alpha-2A adrenergic receptor, insulin, lysophosphatidic acid transferase, glucokinase and PPAR gamma) are known anti-diabetic drug targets of 22 drugs on the market or at clinical trials for diabetic therapeutics (S5 Table), indicating that the current reposition strategy works well and has the potential to reveal novel indications.

In addition, 30 of the 35 proteins with a current indication to treat other diseases may be repurposed to treat diabetes (S6 Table). They correspond to 167 drugs at the approved or clinical trial stages.

### 3.5 Pathogenesis knowledge leads to repositioning 12 targets for treating diabetes

The ‘omics’ results only suggest an association between proteins and risk of diabetes; it does not indicate the cause-effect mechanism. The drugs blocking or activating target proteins usually cause the loss or gain of function of the target. So, we cannot predict the outcome of the drugs without knowing the pathogenesis information of that specific target protein [12]. The current study used the OMIM database and a literature search of human or animal studies to gather knowledge of GOF or LOF for these 30 candidate targets. We excluded 3 targets (CD1a, 5HT 2B, DHOD) corresponding to 11 drugs projects, because they have no direct pathogenesis links to diabetes. We also excluded 6 targets corresponding to 14 drugs that were associated with diabetic complications. According to the drug action mode information from TTD, we excluded 102 drug projects corresponding to 14 targets, since they may aggravate the diabetic symptoms.

Finally, 58 unique drugs corresponding to 12 protein targets had pathogenesis information that supports their therapeutic potential for diabetes (Table 1). Interestingly, one target (MTNR1B) has previously been repurposed for diabetes treatment [25,26]. Another target (Alpha-2A adrenergic receptor) has one drug under phase II clinical trial for diabetes treatment (Yohimbine).

**Table 1:** Information of the 12 targets and 58 drugs repurposed for treating diabetes based on “omics” data mining.

Drug name	Current drug indication	Stage	Target	Action mode	Pathogenesis
**Phenoxybenzamine**	Hypertension, hypoplastic left heart syndrome	Approval	Alpha-2A adrenergic receptor	Antagonist	LOF, rescue insulin secretion in congenic islets^#^
**Idazoxan**	Major Depressive Disorder	Phase III withdraw	Alpha-2A adrenergic receptor	Antagonist	LOF, rescue insulin secretion in congenic islets^#^
**Clobetasol**	Inflammation and itching	Approved	Phospholipase A2	Inhibitor	GOF, deleterious to normal beta-cell secretory function^15^
**Desonide**	Atopic dermatitis	Approved	Phospholipase A2	Inhibitor	GOF, deleterious to normal beta-cell secretory function^15^
**Desoximetasone**	Inflammatory diseases	Approved	Phospholipase A2	Inhibitor	GOF, deleterious to normal beta-cell secretory function^15^
**Diflorasone**	Skin Allergies	Approved	Phospholipase A2	Inhibitor	GOF, deleterious to normal beta-cell secretory function^15^
**Halobetasol Propionate**	Inflammatory diseases	Approved	Phospholipase A2	Inhibitor	GOF, deleterious to normal beta-cell secretory function^15^
**Hydrocortamate**	Inflammatory diseases	Approved	Phospholipase A2	Inhibitor	GOF, deleterious to normal beta-cell secretory function^15^
**Quinacrine**	Giardiasis and cutaneous leishmaniasis	Approved	Phospholipase A2	Inhibitor	GOF, deleterious to normal beta-cell secretory function^15^
**Miltefosine**	Visceral Leishmaniasis, Fungal diseases	Phase II	Phospholipase A2	Inhibitor	GOF, deleterious to normal beta-cell secretory function^15^
**Varespladib**	Coronary Artery Disease, Atherosclerosis	Phase II	Phospholipase A2	Inhibitor	GOF, deleterious to normal beta-cell secretory function^15^
**Echothiophate Iodide**	Chronic glaucoma	Approved	Cholinesterase	Inhibitor	LOF, potentiate insulin action^16^
**Hexafluronium bromide**	Spasms, Pain	Approved	Cholinesterase	Inhibitor	LOF, potentiate insulin action^16^
**Hydrocortisone**	Inflammatory diseases	Approved	Nitric oxide synthase, inducible	Inhibitor	GOF, impair beta-cell function^#^; LOF, reversed fasting hyperglycemia^17^
**Carprofen**	Pain	Approved	Prostaglandin G/H synthase 2	Inhibitor	LOF, increase insulin secretion^18^; GOF, IDDM^19^
**Celecoxib**	Rheumatoid arthritis and osteoarthritis	Approved	Prostaglandin G/H synthase 2	Inhibitor	LOF, increase insulin secretion^18^; GOF, IDDM^19^
**Diflunisal**	Pain	Approved	Prostaglandin G/H synthase 2	Inhibitor	LOF, increase insulin secretion^18^; GOF, IDDM^19^
**Etodolac**	Pain	Approved	Prostaglandin G/H synthase 2	Inhibitor	LOF, increase insulin secretion^18^; GOF, IDDM^19^
**Etoricoxib**	Rheumatoid arthritis and osteoarthritis	Approved	Prostaglandin G/H synthase 2	Inhibitor	LOF, increase insulin secretion^18^; GOF, IDDM^19^
**Ibuprofen**	Pain	Approved	Prostaglandin G/H synthase 2	Inhibitor	LOF, increase insulin secretion^18^; GOF, IDDM^19^
**Ketoprofen**	Rheumatoid arthritis and pain	Approved	Prostaglandin G/H synthase 2	Inhibitor	LOF, increase insulin secretion^18^; GOF, IDDM^19^
**Lumiracoxib**	Knee osteoarthritis	Approved	Prostaglandin G/H synthase 2	Inhibitor	LOF, increase insulin secretion^18^; GOF, IDDM^19^
**Mefenamic acid**	Rheumatoid arthritis and osteoarthritis	Approved	Prostaglandin G/H synthase 2	Inhibitor	LOF, increase insulin secretion^18^; GOF, IDDM^19^
**Meloxicam**	Arthritis	Approved	Prostaglandin G/H synthase 2	Inhibitor	LOF, increase insulin secretion^18^; GOF, IDDM^19^
**Nabumetone**	Rheumatoid arthritis and osteoarthritis	Approved	Prostaglandin G/H synthase 2	Inhibitor	LOF, increase insulin secretion^18^; GOF, IDDM^19^
**Naproxen**	Pain and Rheumatoid arthritis	Approved	Prostaglandin G/H synthase 2	Inhibitor	LOF, increase insulin secretion^18^; GOF, IDDM^19^
**Niflumic Acid**	Rheumatoid arthritis	Approved	Prostaglandin G/H synthase 2	Inhibitor	LOF, increase insulin secretion^18^; GOF, IDDM^19^
**Phenylbutazone**	Chronic pain	Approved	Prostaglandin G/H synthase 2	Inhibitor	LOF, increase insulin secretion^18^; GOF, IDDM^19^
**Piroxicam**	Pain	Approved	Prostaglandin G/H synthase 2	Inhibitor	LOF, increase insulin secretion^18^; GOF, IDDM^19^
**Tenoxicam**	Rheumatoid arthritis and osteoarthritis	Approved	Prostaglandin G/H synthase 2	Inhibitor	LOF, increase insulin secretion^18^; GOF, IDDM^19^
**Tiaprofenic acid**	Pain	Approved	Prostaglandin G/H synthase 2	Inhibitor	LOF, increase insulin secretion^18^; GOF, IDDM^19^
**Tolmetin**	Rheumatoid arthritis and osteoarthritis	Approved	Prostaglandin G/H synthase 2	Inhibitor	LOF, increase insulin secretion^18^; GOF, IDDM^19^
**Valdecoxib**	Osteoarthritis and rheumatoid arthritis	Approved	Prostaglandin G/H synthase 2	Inhibitor	LOF, increase insulin secretion^18^; GOF, IDDM^19^
**ONO-2506**	Stroke	Phase III completed	Prostaglandin G/H synthase 2	Inhibitor	LOF, increase insulin secretion^18^; GOF, IDDM^19^
**Celecoxib**	Pain	Phase III	Prostaglandin G/H synthase 2	Inhibitor	LOF, increase insulin secretion^18^; GOF, IDDM^19^
**GSK-644784**	Neuropathic pain	Suspended in Phase II	Prostaglandin G/H synthase 2	Inhibitor	LOF, increase insulin secretion^18^; GOF, IDDM^19^
**GW-406381**	Osteoarthritis, Neuropathic pain	Suspended in Phase III	Prostaglandin G/H synthase 2	Inhibitor	LOF, increase insulin secretion^18^; GOF, IDDM^19^
**Rofecoxib**	Osteoarthritis	Withdrawn	Prostaglandin G/H synthase 2	Inhibitor	LOF, increase insulin secretion^18^; GOF, IDDM^19^
**D-cycloserine**	Bacterial infections	Approved	NMDA receptor	Agonist	GOF, lower glucose production^20^
**D-cycloserine**	Obsessive-compulsive disorder	Phase II	NMDA receptor	Agonist	GOF, lower glucose production^20^
**D-serine**	Schizophrenia	Phase II	NMDA receptor	Agonist	GOF, lower glucose production^20^
**Remacemide**	Parkinson's Disease	Discontinued in Phase I	NMDA receptor	Agonist	GOF, lower glucose production^20^
**Remacemide**	Huntington's disease	Discontinued in Phase III	NMDA receptor	Agonist	GOF, lower glucose production^20^
**Buspirone**	Anxiety disorder	Approved	Serotonin-1A	Agonist	LOF, impair insulin secretion^21^
**Flibanserin**	Female sexual dysfunction	Phase III	Serotonin-1A	Agonist	LOF, impair insulin secretion^21^
**MN-305**	Severe Mood disorder	Phase II	Serotonin-1A	Agonist	LOF, impair insulin secretion^21^
**OPC-14523**	Bulimia nervosa OCD MDD, severe mood disorders	Phase II	Serotonin-1A	Agonist	LOF, impair insulin secretion^21^
**TGBA01AD**	Severe Mood disorder	Phase II	Serotonin-1A	Agonist	LOF, impair insulin secretion^21^
**OPC-14523**	Female sexual dysfunction	Phase I	Serotonin-1A	Agonist	LOF, impair insulin secretion^21^
**1192U90**	Schizophrenia	Discontinued	Serotonin-1A	Agonist	LOF, impair insulin secretion^21^
**Adatanserin**	Severe Mood disorder	Discontinued in phase II	Serotonin-1A	Agonist	LOF, impair insulin secretion^21^
**Bifeprunox**	Schizophrenia	Teminated in phase III	Serotonin-1A	Agonist	LOF, impair insulin secretion^21^
**PRX-00023**	Severe Mood disorder	Discontinued in phase II	Serotonin-1A	Agonist	LOF, impair insulin secretion^21^
**SLV-313**	Schizophrenia	Terminated in phase I	Serotonin-1A	Agonist	LOF, impair insulin secretion^21^
**Sarizotan**	Parkinson's Disease	Discontinued in phase II	Serotonin-1A	Agonist	LOF, impair insulin secretion^21^
**Perhexiline**	Angina pectoris	Approved	Carnitine O-palmitoyltransferase I	Inhibitor	LOF, decrease glucose production^#^ and reduce insulin resistance^22^
**Cisapride**	Gastroesophageal	Approved	5-hydroxytryptamine 4 receptor	Agonist	GOF, improve insulin sensitivity^23^
**Medusa IL-2**	Cancer/Tumors	Phase I/II	Interleukin-2 receptor subunit beta	Agonist	GOF, reverse/prevent diabetes^24^
**Sotrastaurin acetate**	Renal Transplant	Phase II	Protein kinase C, theta type	Inhibitor	LOF, prevent insulin resistance^#^
**Ramelteon**	Insomnia	Approved	Melatonin receptor type 1B*	Agonist	LOF, type 2 diabetes^#^

* Melatonin receptor type 1B was previously repurposed as a target for diabetes treatment.

# Information was retrieved from OMIM.

### 3.6 CMap results support ‘omics data’ based repositioned drugs

CMap was previously used successfully for drug repositioning by measuring the similarity in gene expression profiles between compounds in mammalian cell lines. The current study analyzed 58 drugs by CMap and assessed their association with known anti-diabetic drugs or diabetic risk compounds. We found that 9 of the 58 drugs (15.5%) have CMap information relating to anti-diabetic drugs or diabetic risk compounds (S7 Table), 11 of the 58 (19.0%) drugs have CMap data but lack links to anti-diabetic drugs or diabetic risk compounds, and 38 of the 58 (65.5%) drugs have no CMap information.

CMap analysis further indicated 9 repurposed drugs for diabetes treatment. Specifically, phenoxybenzamine (enrichment score = 0.799, p = 0.034), nabumetone (enrichment score = 0.576, p = 0.02), niflumic acid (enrichment score = 0.484, p = 0.018) and perhexiline (enrichment score = 0.697, p = 0.00006) are associated with resveratrol that is known to improve glucose metabolism in animals. Idazoxan (enrichment score = 0.728, p = 0.011) and d-cycloserine (enrichment score = 0.56, p = 0.036) are positively associated with gliclazide (an anti-diabetic drug). And diflorasone (enrichment score = -0.709, p = 0.015) is inversely associated with streptozocin that is known to induce diabetes in animals. Diflunisal (enrichment score = 0.626, p = 0.049) is associated with glimepiride (a sulfonylurea anti-diabetic drug), and valdecoxib (enrichment score = 0.412, p = 0.047) is associated with metformin that is well known for treating diabetes.

## Discussion

Using ‘omics’ data mining and pathogenesis information, the current study repurposed 58 drugs for potential diabetes treatment. Gene expression profile comparison indicated 9 drugs with a higher potential in treating diabetes.

**Fig 3:**
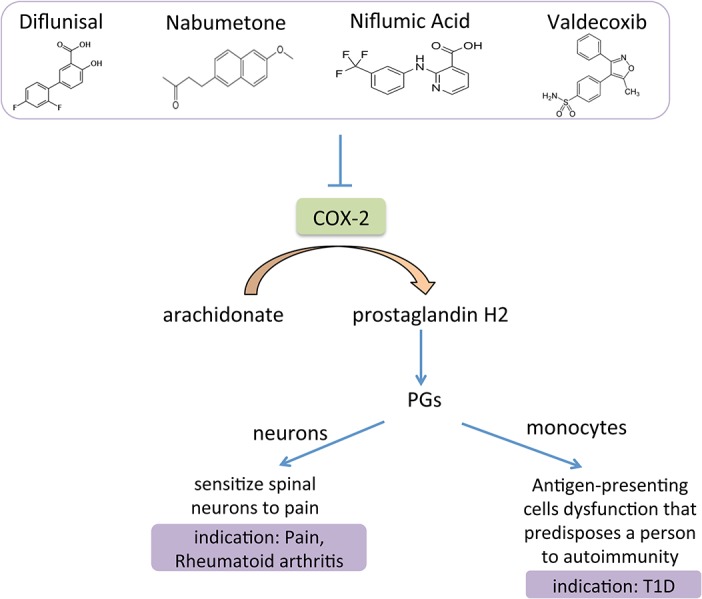
Diagram of COX2 inhibitors and their indication for treating type 1 diabetes. COX2 is known to convert arachidonate to PG H2, the precursor of PGs. PGs have an inflammation effect and sensitize neurons to pain or induce antigen-presenting cell dysfunction that predisposes a person to autoimmunity and type 1 diabetes. Therefore, inhibitors of COX2 have the potential to block PGs-mediated autoimmunity and treat type 1 diabetes.

Among these 9 drugs, diflunisal, nabumetone, niflumic acid and valdecoxib have a common target of prostaglandin G/H synthase 2 (COX2). COX2 converts arachidonate to prostaglandin H2 *in vivo*. Importantly, arachidonate was reported to be increased in the serum of type 2 diabetes and gestational diabetes patients [27,28]. Moreover, LOF of COX2 may increase insulin secretion, and GOF of COX2 may induce insulin dependent diabetes mellitus (IDDM) [18,19]. Similar to other inhibitors of COX2, diflunisal is currently used for pain treatment, and nabumetone, niflumic acid and valdecoxib are used for treating rheumatoid arthritis and osteoarthritis. Importantly, CMap analysis showed that mammalian cells treated by these 4 drugs have a similar gene expression pattern as cells treated by anti-diabetic drugs (glimepiride and metformin) or resveratrol (an activator of Sirt1 and PGC1a, previously shown to improve glucose metabolism) [29]. These evidences collectively indicate that COX2 could be a potential drug target for type 1 diabetes (also called IDDM) treatment. Therefore, the COX2 inhibitors are promising candidates for treating diabetes due to their ability to block prostaglandins (PGs) formation in monocytes and prevent antigen-presenting cell dysfunction, both of which could predispose a person to autoimmunity and IDDM (Fig 3). Interestingly, a Phase II clinical trial (ClinicalTrials.gov Identifier: NCT00506298) was conducted to assess the efficacy of CRx-401 (bezafibrate + diflunisal vs bezafibate + placebo) in lowering fasting plasma glucose levels in patients taking metformin, but the results were not revealed.

Among the 9 drugs, phenoxybenzamine and Idazoxan target ADRA2A (alpha-2A adrenergic receptor). A genetic variation of ADRA2A (risk A allele for rs553668) is known to cause overexpression of ADRA2A, which aggravates adrenergic suppression of insulin secretion and causes type 2 diabetes [30]. Therefore, ADRA2A inhibitors may be utilized to treat a subset of type 2 diabetes patients who carry the genetic risk variant in ADRA2A gene. In fact, one ADRA2A inhibitor drug (Yohimbine) is under phase II clinical trial for treating type 2 diabetes (clinicaltrial.org identifier: NCT01593215).

The other 3 promising drugs are diflorasone, d-cycloserine and perhexiline. Diflorasone inhibits phospholipase A2, a protein previously shown to generate arachidonic acid [27,28] and disrupt beta cell insulin stores [15]. Therefore, diflorasone has the potential to improve beta cell function. Glycine, a co-agonist of the NMDA receptor, was shown to be reduced in type 2 diabetes or gestational diabetes patients in 4 independent studies (S4 Table). Activation of NMDA receptors in the brain by d-cycloserine (NMDA receptor agonist) may have the potential to reduce glucose production and treat diabetes [20]. Perhexiline is an inhibitor of carnitine O-palmitoyltransferase I that converts carnitine (reduced in diabetes condition) to acyl-carnitine (increased in diabetes condition) during lipid beta oxidation. Perhexiline has the potential to improve insulin sensitivity and treat type 2 diabetes because inhibition of carnitine palmitoyltransferase-1 activity was reported to alleviate insulin resistance in diet-induced obese mice [22].

CMap results should be taken with cautious, since this technique has a “batch effect” [31]. For example, cells under the same culture conditions after different compound treatment may have highly similar expression patterns. A strategy of calculating Bridge Adjusted Expression Similarity (BAES) [32] to improve data quality may be used to minimize the batch effect in the future.

The current study integrates biomolecular information associated with diabetes from genomics, proteomics and metabolomics studies. A network of diabetic metabolites and proteins was generated to give an overview of how diabetes alters metabolites. This map may be used to identify the dysfunctional metabolic enzymes in diabetic patients. As diabetes is a metabolic disorder caused by both genetic and environmental factors, analyzing genome data together with protein/metabolite data could provide an in-depth understanding of diabetic etiology.

In terms of the origins of the proposed 9 drugs (S7 Table), 7 were discovered from metabolomics studies and 2 were repurposed from GWAS studies, indicating that the GWAS and metabolomics results provided the most valuable dataset for anti-diabetic drug repositioning in the current study. Interestingly, one of the most successful compound anti-diabetic drugs, metformin, targets a metabolic enzyme (ACC2). The current study is the first to include metabolomics data into drug repositioning, which may assist in the identification of dysfunctional metabolic enzymes or transporters underlying the altered metabolic profiles.

Mapping diabetes risk proteins to drug projects is a critical step in drug repositioning. In the current study, the well-known public Therapeutic Target Database (version 4.3.02) containing information on 236 targets and 20667 drugs was used to perform the mapping. In future studies, other databases such as DrugBank (http://www.drugbank.ca) may also be used to obtain additional information for disease related proteins and to validate initial findings.

In summary, drug repositioning through mining ‘omics’ data provides a powerful tool to find novel indications for marketed drugs and clinical candidates of complex human diseases, such as diabetes. By analyzing GWAS, proteomic and metabolomic data in diabetes, mapping diabetes related proteins to drug projects (TTD), and inputting pathogenesis knowledge, we repurposed 58 drugs with a potential indication for diabetes treatment. Preclinical or clinical trials might be initiated to establish the efficacy of these repositioned drugs for the purpose of diabetes treatment. Furthermore, the results of our study could be related to other common disorders. For instance, there is convincing evidence of an increased risk of dementia in people with diabetes, including a strong link between type 2 diabetes and Alzheimer's disease [33]. Of note, an anti-diabetic drug (Liraglutide) has been used for a Phase II clinical trial for treating Alzheimer’s disease (ClinicalTrials.gov Identifier: NCT01843075).

## Supporting Information

S1 Table Diabetic drugs at the stages of phase III or beyond in 2013–2014 of leading pharmaceutical companies. (DOCX)

S2 Table GWAS studies revealed 115 genes significantly associated with diabetes, impaired insulin response or fasting glucose. (DOCX)

S3 Table Proteomics studies revealed 58 proteins significantly associated with diabetes. (DOCX)

S4 Table Metabolomics studies revealed 227 metabolites significantly associated with diabetes. (DOCX)

S5 Table 5 targets and 27 drugs have been used for diabetes treatment or at the stage of clinical trials. (DOCX)

S6 Table 30 proteins corresponding to 167 drug projects might be repurposed for novel indications for treating diabetes. (DOCX)

S7 Table CMap analysis of 58 drugs. (DOCX)
