# Supplementary material for: Drug Repositioning for Diabetes Based on 'Omics' Data Mining
Source: PLoS One. 2015 May 6;10(5):e0126082. doi: 10.1371/journal.pone.0126082 (PMC4422696; doi:10.1371/journal.pone.0126082)
Supplement: S1 Table — (DOCX) [file pone.0126082.s001.docx]

**S1 Table**. Diabetic drugs at the stages of phase III or beyond in 2013-2014 of leading pharmaceutical companies

| **Name** | **Description** | **Function** | **Stage** | **Indication** | **Company** |
| --- | --- | --- | --- | --- | --- |
| Dapagliflozin | SGLT2 inhibitor | blocking resorption of glucose in the kidney | Approved in EU, US, Japan in 2014 | T2D | AstraZeneca/Bristol-Myers Squibb |
| Metreleptin | leptin analog | improve insulin sensitivity | Under FDA review | T2D | AstraZeneca/Bristol-Myers Squibb |
| Canagliflozin | SGLT2 inhibitor | blocking resorption of glucose in the kidney | Approved in 2013 | T2D | Johnson & Johnson |
| LY2605541 | insulin analog | Long term insulin effect | Phase III | T1D, T2D | Eli Lilly/Boehringer Ingelheim |
| Empagliflozin | SGLT2 inhibitor | blocking resorption of glucose in the kidney | Close to approval in EU | T2D | Eli Lilly/Boehringer Ingelheim |
| LY2963016 | insulin glargine product | Long term insulin effect | Submitted to FDA in 2013 | T1D, T2D | Eli Lilly/Boehringer Ingelheim |
| Dulaglutide | GLP-1 analog | increase insulin secretion upon high glucose | Phase III | T2D | Eli Lilly/Boehringer Ingelheim |
| MK-3102 | DPP4 inhibitor | slows down the breakdown of incretin hormones | Phase III | T2D | Merck & Co. |
| MK-8835 | SGLT2 inhibitor | blocking resorption of glucose in the kidney | Phase III | T2D | Merck & Co. |
| MK-1293 | insulin glargine product | Long term insulin effect | Phase III | T1D, T2D | Merck & Co./Samsung Bioepis |
| Semaglutide | GLP-1 analog | increase insulin secretion upon high glucose | Phase III | T2D | Novo Nordisk |
| NN1250 | insulin analog | Long term insulin effect | Approved in EU and Japan | T1D, T2D | Novo Nordisk |
| NN5401 | insulin analog | Long term insulin effect | Approved in EU and Japan | T1D, T2D | Novo Nordisk |
| NN9068 | insulin analog and GLP1 analog | Long term insulin effect and GLP-1 effect | Regulatory review | T2D | Novo Nordisk |
| NN1218 | insulin analog | Long term insulin effect | Phase III | T1D, T2D | Novo Nordisk |
| NN9211 | GLP-1 analog | increase insulin secretion upon high glucose | Phase III | T1D | Novo Nordisk |
| Lyxumia | GLP-1 analog | increase insulin secretion upon high glucose | Approved in EU in 2013 | T2D | Sanofi |
| Toujeo | insulin analog | Long term insulin effect | Phase III | T1D, T2D | Sanofi |
| alogliptin | DPP4 inhibitor | slows down the breakdown of incretin hormones | Approved in EU in 2013 | T2D | Takeda |
| SYR-472 | DPP4 inhibitor | slows down the breakdown of incretin hormones | Filled in Japn in 2014 | T2D | Takeda |
| Albiglutide | GLP-1 analog | increase insulin secretion upon high glucose | Approved in EU and US in 2014 | T2D | GSK |
| ertugliflozin | SGLT2 inhibitor | blocking resorption of glucose in the kidney | Phase III | T2D | Pfizer |
| ASP1941 | SGLT2 inhibitor | blocking resorption of glucose in the kidney | Approved in Japan in 2014 | T2D | Astellas |
| nateglinide | potassium ATP channel inhibitor | stimulating the release of insulin from the pancreas. | Phase III | T2D | Astellas |
