# Supplementary material for: Drug Repositioning for Diabetes Based on 'Omics' Data Mining
Source: PLoS One. 2015 May 6;10(5):e0126082. doi: 10.1371/journal.pone.0126082 (PMC4422696; doi:10.1371/journal.pone.0126082)
Supplement: S2 Table — (DOCX) [file pone.0126082.s002.docx]

**S2 Table.** GWAS studies revealed 115 genes significantly associated with diabetes, impaired insulin response or fasting glucose.

| **Risk Gene** | **SNP** | **Ref** | **Ethnicity** | **Phynotype** |
| --- | --- | --- | --- | --- |
| GCC1-PAX4 | rs6467136 | 1 | East Asians | T2D |
| PEPD | rs3786897 | 1 | East Asians | T2D |
| PSMD6 | rs831571 | 1 | East Asians | T2D |
| WWOX | rs17797882 | 1 | East Asians | T2D |
| MAEA | rs6815464 | 1 | East Asians | T2D |
| GLIS3 | rs7041847 | 1 | East Asians | T2D |
| FITM2-R3HDMl-HNF4A | rs6017317 | 1 | East Asians | T2D |
| ZFAND3 | rs9470794 | 1 | East Asians | T2D |
| KCNK16 | rs1535500 | 1 | East Asians | T2D |
| CMIP | rs16955379 | 1 | East Asians | T2D |
| CDKAL1 | rs10946398 | 2 | European | T2D |
| IGF2BP2 | rs4402926 | 2 | European | T2D |
| KCNJ11 | rs5215 | 2 | European | T2D |
| PPARG | rs1801282 | 2 | European | T2D |
| WFS1 | rs10010131 | 2 | European | T2D |
| TCF7L2 | rs7901695 | 2 | European | T2D |
| TCF2 | rs4430796 | 2 | European | T2D |
| HHEX-IDE | rs1111875 | 2 | European | T2D |
| SLC30A8 | rs13266634 | 2 | European | T2D |
| CDKN2A-2B | rs10811661 | 2 | European | T2D |
| FTO | rs8050136 | 2 | European | T2D |
| BCL11A | rs243021 | 3 | European | T2D |
| DUSP9 | rs5945326 | 3 | European | T2D |
| HMGA2 | rs1531343a | 3 | European | T2D |
| HNF1A | rs7957197 | 3 | European | T2D |
| IRS1 | rs7578326 | 3 | European | T2D |
| KCNQ1 | rs231362 | 3 | European | T2D |
| PRC1 | rs8042680 | 3 | European | T2D |
| ZBED3 | rs4457053 | 3 | European | T2D |
| CENTD2 | rs1552224 | 3 | European | T2D |
| CHCHD9 | rs13292136 | 3 | European | T2D |
| KLF14 | rs972283 | 3 | European | T2D |
| MTNR1B | rs1387153 | 3 | European | T2D |
| TP53INP1 | rs896854 | 3 | European | T2D |
| ZFAND6 | rs11634397 | 3 | European | T2D |
| TCF2 | rs757210 | 4 | European | T2D |
| ADAMTS9 | rs4607103 | 5 | European | T2D |
| CDC123/CAMK1D | rs12779790 | 5 | European | T2D |
| NOTCH2 | rs10923931 | 5 | European | T2D |
| TSPAN8/LGR5 | rs7961581 | 5 | European | T2D |
| JAZF1 | rs864745 | 5 | European | T2D |
| THADA | rs7578597 | 5 | European | T2D |
| PTPRD | rs17584499 | 6 | East Asians | T2D |
| SRR | rs4523957 | 6 | East Asians | T2D |
| C2CD4A-C2CD4B | rs7172432/rs1436953 | 7 | East Asians | T2D |
| UBE2E2 | rs6780569/rs7612463 /rs9812056 | 7 | East Asians | T2D |
| ADRA2A | rs10885122 | 8 | European | fasting glucose |
| CRY2 | rs11605924 | 8 | European | fasting glucose |
| DGKB/TMEM195 | rs2191349 | 8 | European | fasting glucose |
| FADS1 | rs174550 | 8 | European | fasting glucose |
| GCK | rs4607517 | 8 | European | fasting glucose |
| GCKR | rs780094 | 8 | European | fasting glucose |
| GLIS3 | rs7034200 | 8 | European | fasting glucose |
| MADD | rs7944584 | 8 | European | fasting glucose |
| PROX1 | rs340874 | 8 | European | fasting glucose |
| IGF1 | rs35767 | 8 | European | fasting glucose |
| G6PC2 | rs560887 | 8 | European | fasting glucose |
| MTNR1B | rs10830963 | 8 | European | fasting glucose |
| ADCY5 | rs11708067 | 8 | European | fasting glucose |
| SLC2A2 | rs11920090 | 8 | European | fasting glucose |
| FAM148B | rs11071657 | 8 | European | fasting glucose |
| SLC30A8 | rs11558471 | 8 | European | fasting glucose |
| TCF7L2 | rs4506565 | 8 | European | fasting glucose |
| VPS13C | rs17271305 | 9 | European | glucose/insulin response |
| GIPR | rs10423928 | 9 | European | glucose/insulin response |
| ADCY5 | rs2877716 | 9 | European | glucose/insulin response |
| RBMS1 | rs7593730 | 10 | European | T2D |
| DNER | rs1861612 | 11 | American Indians | T2D |
| PSMD6 | rs831571 | 12 | Japanese | T2D |
| PEPD | rs3786897 | 12 | Japanese | T2D |
| MIR129-LEP | rs791595 | 13 | Japanese | T2D |
| GPSM1 | rs11787792 | 13 | Japanese | T2D |
| SLC16A13 | rs312457 | 13 | Japanese | T2D |
| KCTD8 | rs4407541 | 14 | Arab | T2D |
| GABRB1 | rs10517178/rs1372491 | 14 | Arab | T2D |
| PRKD1 | rs10144903 | 14 | Arab | T2D |
| PAX4 | rs10229583 | 15 | Chinese | T2D |
| PTPN22 | rs2476601 | 16 | Diverse | T1D |
| RGS1 | rs2209014 | 16 | Diverse | T1D |
| IL10 | rs3024493 | 16 | Diverse | T1D |
| AFF3 | rs6740838 | 16 | Diverse | T1D |
| IFIH1 | rs1990760 | 16 | Diverse | T1D |
| STAT4 | rs6744380 | 16 | Diverse | T1D |
| CTLA4 | rs11571316 | 16 | Diverse | T1D |
| CCR5 | rs17078977 | 16 | Diverse | T1D |
| IL2 | rs6827756 | 16 | Diverse | T1D |
| HLA | rs6916742 | 16 | Diverse | T1D |
| BACH2 | rs597325 | 16 | Diverse | T1D |
| TNFAIP3 | rs1878658 | 16 | Diverse | T1D |
| TAGAP | rs212402 | 16 | Diverse | T1D |
| SKAP2 | rs12533947 | 16 | Diverse | T1D |
| GLIS3 | rs10758593 | 16 | Diverse | T1D |
| IL2RA | rs7090530 | 16 | Diverse | T1D |
| PRKCQ | rs2236380 | 16 | Diverse | T1D |
| INS | rs7928968 | 16 | Diverse | T1D |
| CYP27B1 | rs4760341 | 16 | Diverse | T1D |
| SH2B3 | rs3184504 | 16 | Diverse | T1D |
| GPR183 | rs6491500 | 16 | Diverse | T1D |
| DLK1 | rs941576 | 16 | Diverse | T1D |
| RASGRP1 | rs12908309 | 16 | Diverse | T1D |
| IL27 | rs9924471 | 16 | Diverse | T1D |
| ORMDL3 | rs12150079 | 16 | Diverse | T1D |
| PTPN2 | rs1893217 | 16 | Diverse | T1D |
| CD226 | rs1790575 | 16 | Diverse | T1D |
| TYK2 | rs1051738 | 16 | Diverse | T1D |
| UBASH3A | rs11203203 | 16 | Diverse | T1D |
| IL2RB | rs229526 | 16 | Diverse | T1D |
| LMO7 | rs539514 | 16 | Diverse | T1D |
| EFR3 | rs478222 | 16 | Diverse | T1D |

Reference:

1. Meta-analysis of genome-wide association studies identifies eight new loci for type 2 diabetes in east Asians.

Cho YS, Chen CH, Hu C, et al. Nat Genet. 2011 Dec 11;44(1):67-72. doi: 10.1038/ng.1019.

2. Genome-wide association studies provide new insights into type 2 diabetes aetiology. Frayling TM. Nat Rev Genet. 2007 Sep;8(9):657-62.

3. Twelve type 2 diabetes susceptibility loci identified through large-scale association analysis Benjamin F Voight, Laura J Scott, et al. Nat Genet. Author manuscript; available in PMC 2011 April 21.

4. Evaluation of common variants in the six known maturity-onset diabetes of the young (MODY) genes for association with type 2 diabetes. Winckler W, Weedon MN, et al. Diabetes. 2007 Mar;56(3):685-93.

5. Meta-Analysis of Genome-Wide Association Studies in African Americans Provides Insights into the Genetic Architecture of Type 2 Diabetes

Maggie C. Y. Ng, Daniel Shriner, et al. PLoS Genet. 2014 August; 10(8): e1004517. Published online 2014 August 7. doi: 10.1371/journal.pgen.1004517

6. A genome-wide association study identifies susceptibility variants for type 2 diabetes in Han Chinese. Tsai FJ, Yang CF, Chen CC, et al. PLoS Genet. 2010 Feb 19;6(2):e1000847. doi: 10.1371/journal.pgen.1000847.

7. A genome-wide association study in the Japanese population identifies susceptibility loci for type 2 diabetes at UBE2E2 and C2CD4A-C2CD4B.

Yamauchi T, Hara K, et al. Nat Genet. 2010 Oct;42(10):864-8. doi: 10.1038/ng.660. Epub 2010 Sep 5.

8. New genetic loci implicated in fasting glucose homeostasis and their impact on type 2 diabetes risk. Josée Dupuis, Claudia Langenberg, et al. Nat Genet. Author manuscript; available in PMC 2011 January 11.

9. Genetic variation in GIPR influences the glucose and insulin responses to an oral glucose challenge Richa Saxena, Marie-France Hivertet al. Nat Genet. Nat Genet. 2010 February; 42(2): 142–148.

10. Genetic variants at 2q24 are associated with susceptibility to type 2 diabetes. Qi L, Cornelis MC,et al. Hum Mol Genet. 2010 Jul 1;19(13):2706-15. doi: 10.1093/hmg/ddq156. Epub 2010 Apr 23.

11. A genome-wide association study in American Indians implicates DNER as a susceptibility locus for type 2 diabetes. Hanson RL, Muller YL, et al. Diabetes. 2014 Jan;63(1):369-76. doi: 10.2337/db13-0416. Epub 2013 Oct 7.

12. Replication study for the association of 9 East Asian GWAS-derived loci with susceptibility to type 2 diabetes in a Japanese population. Sakai K, Imamura M, et al. PLoS One. 2013 Sep 25;8(9):e76317. doi: 10.1371/journal.pone.0076317.

13. Genome-wide association study identifies three novel loci for type 2 diabetes. Hara K, Fujita H, Johnson TA,et al. Hum Mol Genet. 2014 Jan 1;23(1):239-46.

14. A genome-wide search for type 2 diabetes susceptibility genes in an extended arab family. Al Safar HS, Cordell HJ, Jet al. Ann Hum Genet. 2013 Nov;77(6):488-503.

15. Genome-wide association study in a Chinese population identifies a susceptibility locus for type 2 diabetes at 7q32 near PAX4

R. C. W. Ma, C. et al. Diabetologia. 2013 June; 56(6): 1291–1305.

16. A genome-wide meta-analysis of six type 1 diabetes cohorts identifies multiple associated loci. Bradfield JP, Qu HQ,et al. PLoS Genet. 2011 Sep;7(9):e1002293. doi: 10.1371/journal.pgen.1002293.
