# Supplementary material for: Drug Repositioning for Diabetes Based on 'Omics' Data Mining
Source: PLoS One. 2015 May 6;10(5):e0126082. doi: 10.1371/journal.pone.0126082 (PMC4422696; doi:10.1371/journal.pone.0126082)
Supplement: S3 Table — (DOCX) [file pone.0126082.s003.docx]

**S3 Table.** Proteomics studies revealed 58 proteins significantly associated with diabetes

| **Protein name** | **ID** | **Alter trend** | **Ref** | **Method** | **Sample** | **Phenotype** |
| --- | --- | --- | --- | --- | --- | --- |
| C-reactive protein | P02741 | Up | 1 | MS-ElisA | Serum | T2D |
| Apolipoprotein E | P02649 | UP | 1 | MS-ElisA | Serum | T2D |
| Leptin | P41159 | UP | 1 | MS-ElisA | Serum | T2D |
| Galectin-1 | P09382 | UP | 2 | 2DE-MS-ElisA | plasma | T2D |
| Ficolin-3 | O75636 | Up | 3 | MS-WB | Serum | T2D |
| Complement component 3 | P01024 | Up | 3 | MS-WB | Serum | T2D |
| C3-a | P01024 | Up | 3 | MS-WB | Serum | T2D |
| C3b alpha chain | P01024 | Up | 3 | MS-WB | Serum | T2D |
| TNF alpha receptor 2 | P20333 | UP | 4 | ElisA | plasma | T2D |
| IL 6 | P08505 | UP | 4 | ElisA | plasma | T2D |
| monocyte chemoattractant protein-1 | P13500 | UP | 5 | ElisA | plasma | T2D |
| Apolipoprotein A1 | P02647 | UP | 6 | MS-MRM | Serum | T2D |
| Afamin | P43652 | UP | 6 | MS-MRM | Serum | T2D |
| VDBP | P02774 | UP | 6 | MS-MRM | Serum | T2D |
| Transthyretin | P02766 | UP | 6 | MS-MRM | Serum | T2D |
| fibrinogen | P02671 | UP | 7 | ELISA | plasma | T2D |
| plasminogen activator inhibitor-1 (PAI-1) | P05121 | UP | 7 | ELISA | plasma | T2D |
| CRP | P02741 | UP | 7 | ELISA | plasma | T2D |
| Adiponectin | Q15848 | Down | 8 | ELISA | Serum | T2D |
| histidine triad nucleotide-binding protein 1 | P49773 | Down | 9 | MALDI-MS | urine | T2D |
| bifunctional aminoacyl-tRNA synthetase | P07814 | Down | 9 | MALDI-MS | urine | T2D |
| clusterin precursor protein | P14018 | Down | 9 | MALDI-MS | urine | T2D |
| α2-macroglobulin | P01023 | UP | 10 | LC-MS/Western | Serum | Diabetes |
| FABP4 | P15090 | UP | 11 | LC-MS/Western | visceral adipose tissues | T2D |
| C1QA | P02745 | UP | 11 | LC-MS/Western | visceral adipose tissues | T2D |
| S100A8 | P05109 | UP | 11 | LC-MS/Western | visceral adipose tissues | T2D |
| SORBS1 | Q9BX66 | UP | 11 | LC-MS/Western | visceral adipose tissues | T2D |
| ACADL | P28330 | DOWN | 11 | LC-MS/Western | visceral adipose tissues | T2D |
| PLIN4 | Q96Q06 | DOWN | 11 | LC-MS/Western | visceral adipose tissues | T2D |
| gelsolin | P06396 | UP | 12 | LC-MSMS | urine | T1D |
| antithrombin-III | P01008 | UP | 12 | LC-MSMS | urine | T1D |
| fibrinogen | P02671 | UP | 13 | LC-MS | plasma | T2D |
| haptoglobin | P00738 | UP | 13 | LC-MS | plasma | T2D |
| vitamin D binding protein | P02774 | DOWN | 13 | LC-MS | plasma | T2D |
| α-1-antitrypsin | P01009 | DOWN | 13 | LC-MS | plasma | T2D |
| transthyretin | P02766 | DOWN | 13 | LC-MS | plasma | T2D |
| apolipoprotein A1 | P02647 | DOWN | 13 | LC-MS | plasma | T2D |
| collagen alpha-2(VI) chain | P12110 | UP | 14 | 2D-DIGE | omental adipose tissue | GDM |
| fibrinogen beta chain | P02675 | UP | 14 | 2D-DIGE | omental adipose tissue | GDM |
| lumican | P51884 | UP | 14 | 2D-DIGE | omental adipose tissue | GDM |
| S100A9 | P06702 | UP | 14 | 2D-DIGE | omental adipose tissue | GDM |
| alpha-1-antitrypsin1 | P02647 | DOWN | 14 | 2D-DIGE | omental adipose tissue | GDM |
| annexin A5 | P08758 | DOWN | 14 | 2D-DIGE | omental adipose tissue | GDM |
| FABP4 | P15090 | DOWN | 14 | 2D-DIGE | omental adipose tissue | GDM |
| GSTP | P09211 | DOWN | 14 | 2D-DIGE | omental adipose tissue | GDM |
| vinculin | P18206 | DOWN | 14 | 2D-DIGE | omental adipose tissue | GDM |
| heat-shock protein beta-1 | P04792 | DOWN | 14 | 2D-DIGE | omental adipose tissue | GDM |
| lactate dehydrogenase B chain | P07195 | DOWN | 14 | 2D-DIGE | omental adipose tissue | GDM |
| perilipin-1 | O60240 | DOWN | 14 | 2D-DIGE | omental adipose tissue | GDM |
| selenium-binding protein 1 | Q13228 | DOWN | 14 | 2D-DIGE | omental adipose tissue | GDM |
| PRDX6 | P30041 | DOWN | 14 | 2D-DIGE | omental adipose tissue | GDM |
| PPBP | P02775 | UP | 15 | LC-MS | serum | T1D |
| plasma protease C1 inhibitor | P05155 | UP | 15 | LC-MS | serum | T1D |
| complement C3 | P01024 | DOWN | 15 | LC-MS | serum | T1D |
| gelsolin | P06396 | DOWN | 15 | LC-MS | serum | T1D |
| N-acetylmuramoyl-L-alanine amidase | Q96PD5 | DOWN | 15 | LC-MS | serum | T1D |
| transthyretin | P02766 | DOWN | 15 | LC-MS | serum | T1D |
| adiponectin | Q15848 | DOWN | 16 | LC-MS | serum | T1D |
| insulin-like growth factor binding protein 2 | P18065 | DOWN | 16 | LC-MS | serum | T1D |
| serum amyloid protein A | P0DJI8 | DOWN | 16 | LC-MS | serum | T1D |
| C-reactive protein | P02741 | DOWN | 16 | LC-MS | serum | T1D |
| myeloperoxidase | P05164 | UP | 16 | LC-MS | serum | T1D |
| transforming growth factor beta induced | P01137 | UP | 16 | LC-MS | serum | T1D |
| Annexin A2 | P07355 | UP | 17 | 2DE-MALDI-TOF | placentas | GDM |
| Annexin A5 | P08758 | UP | 17 | 2DE-MALDI-TOF | placentas | GDM |
| 14-3-3 protein ζ/δ | P63104 | UP | 17 | 2DE-MALDI-TOF | placentas | GDM |
| Ras-related protein Rap1A | P62834 | DOWN | 17 | 2DE-MALDI-TOF | placentas | GDM |

Reference:

1. Proteomic identification of human serum biomarkers in diabetes mellitus type 2.

Riaz S, Alam SS, Akhtar MW. J Pharm Biomed Anal. 2010 Apr 6;51(5):1103-7.

2. Proteomics-based identification of differentially-expressed proteins including galectin-1 in the blood plasma of type 2 diabetic patients.

Liu X, Feng Q, Chen Y, Zuo J, Gupta N, Chang Y, Fang F. J Proteome Res. 2009 Mar;8(3):1255-62.

3. Localized-statistical quantification of human serum proteome associated with type 2 diabetes.

Li RX, Chen HB, et al. PLoS One. 2008 Sep 16;3(9):e3224.

4. Inflammatory markers and risk of developing type 2 diabetes in women. Hu FB, Meigs JB, Li TY, Rifai N, Manson JE.

Diabetes. 2004 Mar;53(3):693-700.

5. Plasma monocyte chemoattractant protein-1 as risk marker in type 2 diabetes mellitus and coronary artery disease in North Indians.

Harsimran K, Singh AA, et al. Diab Vasc Dis Res. 2009 Oct;6(4):288-90.

6. iTRAQ-based quantitative protein expression profiling and MRM verification of markers in type 2 diabetes.

Kaur P, Rizk NM, Ibrahim S, et al. J Proteome Res. 2012 Nov 2;11(11):5527-39. doi: 10.1021/pr300798z.

7. Elevated levels of acute-phase proteins and plasminogen activator inhibitor-1 predict the development of type 2 diabetes: the insulin resistance atherosclerosis study. Festa A, D'Agostino R Jr, Tracy RP, Haffner SM; Insulin Resistance Atherosclerosis Study. Diabetes. 2002 Apr;51(4):1131-7.

8. Clinical implications of adiponectin and inflammatory biomarkers in type 2 diabetes mellitus.

Swellam M, Sayed Mahmoud And M, Abdel-Fatah Ali A. Dis Markers. 2009;27(6):269-78.

9. Identification of urinary biomarkers for type 2 diabetes using bead-based proteomic approach. Chu L, Fu G, Meng Q, Zhou H, Zhang M.

Diabetes Res Clin Pract. 2013 Aug;101(2):187-93.

10. Serum monomeric α2-macroglobulin as a clinical biomarker in diabetes. Takada T, Kodera Y, et al.

Atherosclerosis. 2013 May;228(1):270-6.

11. A protein profile of visceral adipose tissues linked to early pathogenesis of type 2 diabetes mellitus. Kim SJ, Chae S, Kim H, Mun DG, Back S, Choi HY, Park KS, Hwang D, Choi SH, Lee SW. Mol Cell Proteomics. 2014 Mar;13(3):811-22.

12. Pursuing type 1 diabetes mellitus and related complications through urinary proteomics.

Caseiro A, Barros A, Ferreira R, et al. Transl Res. 2014 Mar;163(3):188-99.

13. Proteomic study reveals downregulation of apolipoprotein A1 in plasma of poorly controlled diabetes: a pilot study.

Bhonsle HS, Korwar AM,et al. Mol Med Rep. 2013 Feb;7(2):495-8.

14. 2D-DIGE to identify proteins associated with gestational diabetes in omental adipose tissue. Oliva K, Barker G, Rice GE, Bailey MJ, Lappas M. J Endocrinol. 2013 Jul 1;218(2):165-78.

15. Serum proteomics reveals systemic dysregulation of innate immunity in type 1 diabetes. Zhang Q, Fillmore TL, et al. J Exp Med. 2013 Jan 14;210(1):191-203.

16. Discovery and validation of serum protein changes in type 1 diabetes patients using high throughput two dimensional liquid chromatography-mass spectrometry and immunoassays. Zhi W, Sharma A, Purohit S, et al. Mol Cell Proteomics. 2011 Nov;10(11):M111.012203.

17.Altered protein expression in gestational diabetes mellitus placentas provides insight into insulin resistance and coagulation/fibrinolysis pathways. Liu B, Xu Y, Voss C, et al. PLoS One. 2012;7(9):e44701.
