# Supplementary material for: Drug Repositioning for Diabetes Based on 'Omics' Data Mining
Source: PLoS One. 2015 May 6;10(5):e0126082. doi: 10.1371/journal.pone.0126082 (PMC4422696; doi:10.1371/journal.pone.0126082)
Supplement: S4 Table — (DOCX) [file pone.0126082.s004.docx]

**S4 Table**. Metabolomics studies revealed 227 metabolites significantly associated with diabetes

| **Altered Metabolites** | **Up-down** | **Sample** | **Ethnicity** | **Reference** | **Method** | **Phenotype** |
| --- | --- | --- | --- | --- | --- | --- |
| hexose | Up | Serum | European | 1 | LC-MS | T2D |
| phenylalanine | Up | Serum | European | 1 | LC-MS | T2D |
| PC aa C32:1 | Up | Serum | European | 1 | LC-MS | T2D |
| PC aa C36:1 | Up | Serum | European | 1 | LC-MS | T2D |
| PC aa C38:3 | Up | Serum | European | 1 | LC-MS | T2D |
| PC aa C40:5 | Up | Serum | European | 1 | LC-MS | T2D |
| glycine | Down | Serum | European | 1 | LC-MS | T2D |
| sphingomyelin C16:1 | Down | Serum | European | 1 | LC-MS | T2D |
| PC ae C34:3 | Down | Serum | European | 1 | LC-MS | T2D |
| PC ae C40:6 | Down | Serum | European | 1 | LC-MS | T2D |
| PC ae C42:5 | Down | Serum | European | 1 | LC-MS | T2D |
| PC ae C44:4 | Down | Serum | European | 1 | LC-MS | T2D |
| PC ae C44:5 | Down | Serum | European | 1 | LC-MS | T2D |
| lysophosphatidylcholine C18:2 | Down | Serum | European | 1 | LC-MS | T2D |
| glycine | Down | Serum | European | 2 | LC-MS | T2D/IGT |
| lysophosphatidylcholine (LPC) (18:2) | Down | Serum | European | 2 | LC-MS | T2D/IGT |
| acetylcarnitine | UP | Serum | European | 2 | LC-MS | IGT |
| hexose | UP | Serum | European | 2 | LC-MS | T2D |
| Ile | UP | Serum | European | 2 | LC-MS | T2D |
| PC aa C32:1 | UP | Serum | European | 2 | LC-MS | T2D |
| LPC (17:0) | Down | Serum | European | 2 | LC-MS | T2D/IGT |
| PC ae C34:3 | Down | Serum | European | 2 | LC-MS | T2D |
| PC ae C36:2 | Down | Serum | European | 2 | LC-MS | T2D/IGT |
| PC ae C38:3 | Down | Serum | European | 2 | LC-MS | T2D |
| PC ae C40:3 | Down | Serum | European | 2 | LC-MS | T2D |
| PC ae C40:5 | Down | Serum | European | 2 | LC-MS | T2D |
| SM C16:0 | Down | Serum | European | 2 | LC-MS | T2D |
| SM C16:1 | Down | Serum | European | 2 | LC-MS | T2D |
| SM C20:2 | Down | Serum | European | 2 | LC-MS | T2D |
| SM(OH) C16:1 | Down | Serum | European | 2 | LC-MS | T2D |
| SM (OH) C22:1 | Down | Serum | European | 2 | LC-MS | T2D |
| SM (OH) C22:2 | Down | Serum | European | 2 | LC-MS | T2D |
| SM(OH) C24:1 | Down | Serum | European | 2 | LC-MS | T2D |
| LPC(18:0) | Down | Serum | European | 2 | LC-MS | IGT |
| LPC(18:1) | Down | Serum | European | 2 | LC-MS | IGT |
| PC ae C34:2 | Down | Serum | European | 2 | LC-MS | IGT |
| PC ae C36:3 | Down | Serum | European | 2 | LC-MS | IGT |
| Hippurate | Down | Urine | European | 3 | HNMR | Obese |
| Medium- and short-chain AcylCNs (free CN-C6) | Down | plasma | US | 4 | LC-MS | T2D |
| Leu/Ile | Down | plasma | US | 4 | LC-MS | T2D |
| Val | Down | plasma | US | 4 | LC-MS | T2D |
| Phe | Down | plasma | US | 4 | LC-MS | T2D |
| Met | Down | plasma | US | 4 | LC-MS | T2D |
| Ala | Down | plasma | US | 4 | LC-MS | T2D |
| His | Down | plasma | US | 4 | LC-MS | T2D |
| Arg | Down | plasma | US | 4 | LC-MS | T2D |
| Cit | Down | plasma | US | 4 | LC-MS | T2D |
| Ser | Down | plasma | US | 4 | LC-MS | T2D |
| glycine | Down | plasma | US | 4 | LC-MS | T2D |
| Leusine | UP | plasma | US/european | 5 | LC-MS | T2D |
| Isoleusine | UP | plasma | US/european | 5 | LC-MS | T2D |
| Valine | UP | plasma | US/european | 5 | LC-MS | T2D |
| phenylalanine | UP | plasma | US/european | 5 | LC-MS | T2D |
| tyrosine | UP | plasma | US/european | 5 | LC-MS | T2D |
| glutamytvaline | UP | serum | European | 6 | GC/LC-MS | T2D |
| gamma-glutamylisoleucine | UP | serum | European | 6 | GC/LC-MS | T2D |
| 1,5 AG | Down | serum | European | 6 | GC/LC-MS | T2D |
| desoxyhexose | UP | serum | European | 6 | GC/LC-MS | T2D |
| H3-HNAc2-HANA | UP | serum | European | 6 | GC/LC-MS | T2D |
| HNAC | UP | serum | European | 6 | GC/LC-MS | T2D |
| HNAC-H2-dH | UP | serum | European | 6 | GC/LC-MS | T2D |
| uronic acid | UP | serum | European | 6 | GC/LC-MS | T2D |
| dihexose | UP | serum | European | 6 | GC/LC-MS | T2D |
| mannose | UP | serum | European | 6 | GC/LC-MS | T2D |
| caproate (6:0) | Down | serum | European | 6 | GC/LC-MS | T2D |
| heptanoate (7:0) | Down | serum | European | 6 | GC/LC-MS | T2D |
| petargonate (9:0) | Down | serum | European | 6 | GC/LC-MS | T2D |
| glycerophosphorylcholine | Down | serum | European | 6 | GC/LC-MS | T2D |
| PC ae C20:4 | Down | serum | European | 6 | GC/LC-MS | T2D |
| PC ae C28:4 | Down | serum | European | 6 | GC/LC-MS | T2D |
| PC ae C34:4 | Down | serum | European | 6 | GC/LC-MS | T2D |
| SM C14:0 | Down | serum | European | 6 | GC/LC-MS | T2D |
| SM C22:2 | Down | serum | European | 6 | GC/LC-MS | T2D |
| creatinine | Up | serum | European | 6 | GC/LC-MS | T2D |
| gBHBA | Up | serum | European | 6 | GC/LC-MS | T2D |
| phenylacetylglutanine | Up | serum | European | 6 | GC/LC-MS | T2D |
| phenylalanine | Up | serum | European | 6 | GC/LC-MS | T2D |
| 3-indoxyl sulfate | Up | serum | European | 6 | GC/LC-MS | T2D |
| kynutinine | Up | serum | European | 6 | GC/LC-MS | T2D |
| homocitrulline | Up | serum | European | 6 | GC/LC-MS | T2D |
| Lactate | Down | serum | Chinese | 7 | GC-MS | T2D |
| Butanoate | Up | serum | Chinese | 7 | GC-MS | T2D |
| valine | UP | serum | Chinese | 7 | GC-MS | T2D |
| lysine | Down | serum | Chinese | 7 | GC-MS | T2D |
| Glutamate | UP | serum | Chinese | 7 | GC-MS | T2D |
| Glucuronolactone | Down | serum | Chinese | 7 | GC-MS | T2D |
| C16:0 | UP | serum | Chinese | 7 | GC-MS | T2D |
| urate | UP | serum | Chinese | 7 | GC-MS | T2D |
| C18:1 | UP | serum | Chinese | 7 | GC-MS | T2D |
| C18:0 | UP | serum | Chinese | 7 | GC-MS | T2D |
| Arachidonate | UP | serum | Chinese | 7 | GC-MS | T2D |
| maltose | UP | serum | Chinese | 7 | GC-MS | T2D |
| Octadecanoate | Down | serum | Chinese | 7 | GC-MS | T2D |
| dihomo-linolenate (20:3 n3 or n6) | UP | plasma | Canada | 8 | GC/LC-MS | GDM |
| dihomo-linolenate (20:3 n6) | UP | plasma | Canada | 8 | GC/LC-MS | GDM |
| DHA | UP | plasma | Canada | 8 | GC/LC-MS | GDM |
| EPA | UP | plasma | Canada | 8 | GC/LC-MS | GDM |
| arachidonate | UP | plasma | Canada | 8 | GC/LC-MS | GDM |
| 2-hydroxypalmitate | UP | plasma | Canada | 8 | GC/LC-MS | GDM |
| 3-hydroxyoctanoate | UP | plasma | Canada | 8 | GC/LC-MS | GDM |
| eicosenoate (20:1 n9 or 11) | UP | plasma | Canada | 8 | GC/LC-MS | GDM |
| magarate (17:0) | UP | plasma | Canada | 8 | GC/LC-MS | GDM |
| nonadecanoate (19:0) | UP | plasma | Canada | 8 | GC/LC-MS | GDM |
| stearidonate (18:4n3) | UP | plasma | Canada | 8 | GC/LC-MS | GDM |
| 2-hydroxystearate | UP | plasma | Canada | 8 | GC/LC-MS | GDM |
| CMPF | Up | plasma | Canada | 8 | GC/LC-MS | GDM |
| LPE(20:2) | DOWN | plasma | caucasian | 9 | LC-QTOF/MS, GC-Q/MS and CE-TOF/MS | GDM |
| LPE(20:1) | DOWN | plasma | caucasian | 9 | LC-QTOF/MS, GC-Q/MS and CE-TOF/MS | GDM |
| Trihydroxy-cholestanoyl taurine | DOWN | plasma | caucasian | 9 | LC-QTOF/MS, GC-Q/MS and CE-TOF/MS | GDM |
| LPA(18:2) | DOWN | plasma | caucasian | 9 | LC-QTOF/MS, GC-Q/MS and CE-TOF/MS | GDM |
| LPC(20:5) sn-2 | DOWN | plasma | caucasian | 9 | LC-QTOF/MS, GC-Q/MS and CE-TOF/MS | GDM |
| LPI(20:4) | DOWN | plasma | caucasian | 9 | LC-QTOF/MS, GC-Q/MS and CE-TOF/MS | GDM |
| LPC(18:2) sn-2 | DOWN | plasma | caucasian | 9 | LC-QTOF/MS, GC-Q/MS and CE-TOF/MS | GDM |
| PC(21:1) | DOWN | plasma | caucasian | 9 | LC-QTOF/MS, GC-Q/MS and CE-TOF/MS | GDM |
| LPC(18:1) sn-2 | DOWN | plasma | caucasian | 9 | LC-QTOF/MS, GC-Q/MS and CE-TOF/MS | GDM |
| LPE(22:4) | DOWN | plasma | caucasian | 9 | LC-QTOF/MS, GC-Q/MS and CE-TOF/MS | GDM |
| LPS(20:0) | DOWN | plasma | caucasian | 9 | LC-QTOF/MS, GC-Q/MS and CE-TOF/MS | GDM |
| Lipoxin C4 | DOWN | plasma | caucasian | 9 | LC-QTOF/MS, GC-Q/MS and CE-TOF/MS | GDM |
| LPC(22:5) sn-2 | DOWN | plasma | caucasian | 9 | LC-QTOF/MS, GC-Q/MS and CE-TOF/MS | GDM |
| LPC(22:4) sn-2 | DOWN | plasma | caucasian | 9 | LC-QTOF/MS, GC-Q/MS and CE-TOF/MS | GDM |
| LPI(18:2) | DOWN | plasma | caucasian | 9 | LC-QTOF/MS, GC-Q/MS and CE-TOF/MS | GDM |
| LPC(20:2) sn-2 | DOWN | plasma | caucasian | 9 | LC-QTOF/MS, GC-Q/MS and CE-TOF/MS | GDM |
| LPC(20:4) sn-2 | DOWN | plasma | caucasian | 9 | LC-QTOF/MS, GC-Q/MS and CE-TOF/MS | GDM |
| Taurolithocholic acid glucuronide | DOWN | plasma | caucasian | 9 | LC-QTOF/MS, GC-Q/MS and CE-TOF/MS | GDM |
| LPC(19:1) | DOWN | plasma | caucasian | 9 | LC-QTOF/MS, GC-Q/MS and CE-TOF/MS | GDM |
| Glycerophosphocholine | DOWN | plasma | caucasian | 9 | LC-QTOF/MS, GC-Q/MS and CE-TOF/MS | GDM |
| Docosahexaenoic acid methylester | DOWN | plasma | caucasian | 9 | LC-QTOF/MS, GC-Q/MS and CE-TOF/MS | GDM |
| LPI(22:6) | DOWN | plasma | caucasian | 9 | LC-QTOF/MS, GC-Q/MS and CE-TOF/MS | GDM |
| Arachidonic acid methylester | DOWN | plasma | caucasian | 9 | LC-QTOF/MS, GC-Q/MS and CE-TOF/MS | GDM |
| LPC(22:6) sn-2 | DOWN | plasma | caucasian | 9 | LC-QTOF/MS, GC-Q/MS and CE-TOF/MS | GDM |
| LPI(20:3) | DOWN | plasma | caucasian | 9 | LC-QTOF/MS, GC-Q/MS and CE-TOF/MS | GDM |
| LPE(18:2) | DOWN | plasma | caucasian | 9 | LC-QTOF/MS, GC-Q/MS and CE-TOF/MS | GDM |
| LPE(20:4) | DOWN | plasma | caucasian | 9 | LC-QTOF/MS, GC-Q/MS and CE-TOF/MS | GDM |
| LPE(22:6) | DOWN | plasma | caucasian | 9 | LC-QTOF/MS, GC-Q/MS and CE-TOF/MS | GDM |
| Creatinine | DOWN | plasma | caucasian | 9 | LC-QTOF/MS, GC-Q/MS and CE-TOF/MS | GDM |
| Pyruvic acid | DOWN | plasma | caucasian | 9 | LC-QTOF/MS, GC-Q/MS and CE-TOF/MS | GDM |
| L-tryptophan | DOWN | plasma | caucasian | 9 | LC-QTOF/MS, GC-Q/MS and CE-TOF/MS | GDM |
| 2-hydroxybutyric acid | UP | plasma | caucasian | 9 | LC-QTOF/MS, GC-Q/MS and CE-TOF/MS | GDM |
| Glycine | DOWN | plasma | caucasian | 9 | LC-QTOF/MS, GC-Q/MS and CE-TOF/MS | GDM |
| L-glutamic acid | DOWN | plasma | caucasian | 9 | LC-QTOF/MS, GC-Q/MS and CE-TOF/MS | GDM |
| Lauric acid | DOWN | plasma | caucasian | 9 | LC-QTOF/MS, GC-Q/MS and CE-TOF/MS | GDM |
| Glycerol | UP | plasma | caucasian | 9 | LC-QTOF/MS, GC-Q/MS and CE-TOF/MS | GDM |
| 3-hydroxybutyric acid | UP | plasma | caucasian | 9 | LC-QTOF/MS, GC-Q/MS and CE-TOF/MS | GDM |
| Linoleic acid | UP | plasma | caucasian | 9 | LC-QTOF/MS, GC-Q/MS and CE-TOF/MS | GDM |
| Fumaric acid | UP | plasma | caucasian | 9 | LC-QTOF/MS, GC-Q/MS and CE-TOF/MS | GDM |
| Carnitine | DOWN | urine | caucasian | 9 | LC-QTOF/MS, GC-Q/MS and CE-TOF/MS | GDM |
| Histidine | UP | urine | caucasian | 9 | LC-QTOF/MS, GC-Q/MS and CE-TOF/MS | GDM |
| 2-aminoadipic acid | UP | plasma | US and Sweden | 10 | HILIC and LC-MS | T2D |
| Adiponectin | UP | urine | Chinese | 11 | LC-ESI/quadrupole-TOF | T2D |
| acylcarnitines | UP | urine | Chinese | 11 | LC-ESI/quadrupole-TOF | T2D |
| citric acid | DOWN | urine | Chinese | 11 | LC-ESI/quadrupole-TOF | T2D |
| kynurenic acid | DOWN | urine | Chinese | 11 | LC-ESI/quadrupole-TOF | T2D |
| 3-indoxyl sulfate | UP | urine | Chinese | 11 | LC-ESI/quadrupole-TOF | T2D |
| urate | DOWN | urine | Chinese | 11 | LC-ESI/quadrupole-TOF | T2D |
| 3-methyl-2-oxovalerate | UP | Plasma | UK | 12 | GC/LC-MS | T2D/IFG |
| AHB (2-hydroxybutyrate ) | Up | Plasma | UK | 12 | GC/LC-MS | T2D/IFG |
| N-acetylglycine | DOWN | Plasma | UK | 12 | GC/LC-MS | T2D/IFG |
| Citrulline | DOWN | Plasma | UK | 12 | GC/LC-MS | T2D/IFG |
| Dimethylarginine | DOWN | Plasma | UK | 12 | GC/LC-MS | T2D/IFG |
| Proline | UP | Plasma | UK | 12 | GC/LC-MS | T2D/IFG |
| 3-methyl-2-oxobutyrate | UP | Plasma | UK | 12 | GC/LC-MS | T2D/IFG |
| 4-Methyl-2-oxopentanoate | UP | Plasma | UK | 12 | GC/LC-MS | T2D/IFG |
| Isoleucine | UP | Plasma | UK | 12 | GC/LC-MS | T2D/IFG |
| Leucine | UP | Plasma | UK | 12 | GC/LC-MS | T2D/IFG |
| Valine | UP | Plasma | UK | 12 | GC/LC-MS | T2D/IFG |
| Fructose | UP | Plasma | UK | 12 | GC/LC-MS | T2D/IFG |
| Mannose | UP | Plasma | UK | 12 | GC/LC-MS | T2D/IFG |
| 1,5-AG | DOWN | Plasma | UK | 12 | GC/LC-MS | T2D/IFG |
| Lactate | UP | Plasma | UK | 12 | GC/LC-MS | T2D/IFG |
| Arabinose | UP | Plasma | UK | 12 | GC/LC-MS | T2D/IFG |
| Malate | UP | Plasma | UK | 12 | GC/LC-MS | T2D/IFG |
| Octanoylcarnitine | DOWN | Plasma | UK | 12 | GC/LC-MS | T2D/IFG |
| 1,5-Methylpalmitate | DOWN | Plasma | UK | 12 | GC/LC-MS | T2D/IFG |
| 10-Heptadecenoate | DOWN | Plasma | UK | 12 | GC/LC-MS | T2D/IFG |
| Adrenate | UP | Plasma | UK | 12 | GC/LC-MS | T2D/IFG |
| Arachidonate | UP | Plasma | UK | 12 | GC/LC-MS | T2D/IFG |
| Myristate (14:0) | DOWN | Plasma | UK | 12 | GC/LC-MS | T2D/IFG |
| Myristoleate (14:1n5) | DOWN | Plasma | UK | 12 | GC/LC-MS | T2D/IFG |
| Palmitoleate (16:1n7) | DOWN | Plasma | UK | 12 | GC/LC-MS | T2D/IFG |
| Pentadecanoate (15:0) | DOWN | Plasma | UK | 12 | GC/LC-MS | T2D/IFG |
| 5-Dodecenoate (12:1n7) | DOWN | Plasma | UK | 12 | GC/LC-MS | T2D/IFG |
| Hepatanoate (7:0) | DOWN | Plasma | UK | 12 | GC/LC-MS | T2D/IFG |
| Pelargonate (9:0) | DOWN | Plasma | UK | 12 | GC/LC-MS | T2D/IFG |
| Plamitoyl sphingomyelin | DOWN | Plasma | UK | 12 | GC/LC-MS | T2D/IFG |
| Xcholesterol | DOWN | Plasma | UK | 12 | GC/LC-MS | T2D/IFG |
| Urate | UP | Plasma | UK | 12 | GC/LC-MS | T2D/IFG |
| β-hydroxybutyrate | UP | Plasma | US | 13 | QTRAP triple quadrupole MS | IR during OGGT |
| isoleucine | UP | Plasma | US | 13 | QTRAP triple quadrupole MS | IR during OGGT |
| lactate | DOWN | Plasma | US | 13 | QTRAP triple quadrupole MS | IR during OGGT |
| orotate | DOWN | Plasma | US | 13 | QTRAP triple quadrupole MS | IR during OGGT |
| pyridoxate | UP | Plasma | US | 13 | QTRAP triple quadrupole MS | IR during OGGT |
| 3,7,12-Trioxochola-1,4-dien-24-oic acid | DOWN | Plasma | Spain | 13 | LC-MS | T1D |
| Deoxycholic acid 3-glucuronide | UP | Plasma | Spain | 14 | LC-MS | T1D |
| Palmitic acid (C16:0) | UP | Plasma | Spain | 14 | LC-MS | T1D |
| Palmitoleic acid (C16:1ω9) | UP | Plasma | Spain | 14 | LC-MS | T1D |
| Oleic acid (C18:1ω9) | UP | Plasma | Spain | 14 | LC-MS | T1D |
| Linoleic acid (C18:2ω6) | UP | Plasma | Spain | 14 | LC-MS | T1D |
| Linolenic acid (C18:3ω3) | UP | Plasma | Spain | 14 | LC-MS | T1D |
| Adrenic acid (C22:4ω6) | UP | Plasma | Spain | 14 | LC-MS | T1D |
| PC (17:0/2:0) | UP | Plasma | Spain | 14 | LC-MS | T1D |
| LysoPC (14:0) | DOWN | Plasma | Spain | 14 | LC-MS | T1D |
| LysoPE (22:6) | UP | Plasma | Spain | 14 | LC-MS | T1D |
| LysoPE (O-18:1) | UP | Plasma | Spain | 14 | LC-MS | T1D |
| LysoPE (P-16:0) | UP | Plasma | Spain | 14 | LC-MS | T1D |
| 2-Hydroxy capric acid | DOWN | Plasma | Spain | 14 | LC-MS | T1D |
| 11,12-Dihydroxy arachidic acid | UP | Plasma | Spain | 14 | LC-MS | T1D |
| Lysinea | UP | Urine | Spain | 14 | GC-MS | T1D |
| Valinea | UP | Urine | Spain | 14 | GC-MS | T1D |
| Leucine/isoleucinea | UP | Urine | Spain | 14 | GC-MS | T1D |
| Acetylarginine | UP | Urine | Spain | 14 | GC-MS | T1D |
| fructose | UP | Plasma | Singapore | 15 | GC/LC-MS | T2D/IFG |
| α-hydroxybutyrate | UP | Plasma | Singapore | 15 | GC/LC-MS | T2D/IFG |
| alanine | UP | Plasma | Singapore | 15 | GC/LC-MS | T2D/IFG |
| proline | UP | Plasma | Singapore | 15 | GC/LC-MS | T2D/IFG |
| phenylalanine | UP | Plasma | Singapore | 15 | GC/LC-MS | T2D/IFG |
| glutamine | UP | Plasma | Singapore | 15 | GC/LC-MS | T2D/IFG |
| leucine | UP | Plasma | Singapore | 15 | GC/LC-MS | T2D/IFG |
| isoleucine | UP | Plasma | Singapore | 15 | GC/LC-MS | T2D/IFG |
| valine | UP | Plasma | Singapore | 15 | GC/LC-MS | T2D/IFG |
| myristic | UP | Plasma | Singapore | 15 | GC/LC-MS | T2D/IFG |
| palmitic | UP | Plasma | Singapore | 15 | GC/LC-MS | T2D/IFG |
| stearic acid | UP | Plasma | Singapore | 15 | GC/LC-MS | T2D/IFG |
| LysoPC(16:0)d | DOWN | Serum | Chinese | 16 | GC/LC-MS/EISA | T2D/IPD (isolated postchallenge diabetes) |
| LysoPC(18:2)d | DOWN | Serum | Chinese | 16 | GC/LC-MS/EISA | T2D/IPD (isolated postchallenge diabetes) |
| LysoPC(18:0)d | DOWN | Serum | Chinese | 16 | GC/LC-MS/EISA | T2D/IPD (isolated postchallenge diabetes) |
| Docosanoic acidd | UP | Serum | Chinese | 16 | GC/LC-MS/EISA | T2D/IPD (isolated postchallenge diabetes) |
| Cholesteryl-β-D-glucosidee | UP | Serum | Chinese | 16 | GC/LC-MS/EISA | T2D/IPD (isolated postchallenge diabetes) |
| Cholesteryl-β-D-glucoside fragmente | UP | Serum | Chinese | 16 | GC/LC-MS/EISA | T2D/IPD (isolated postchallenge diabetes) |
| 1,2-Distearoyl phosphatidyl serinee | UP | Serum | Chinese | 16 | GC/LC-MS/EISA | T2D/IPD (isolated postchallenge diabetes) |
| Linoleic acidd | UP | Serum | Chinese | 16 | GC/LC-MS/EISA | T2D/IPD (isolated postchallenge diabetes) |
| Oleic acidd | UP | Serum | Chinese | 16 | GC/LC-MS/EISA | T2D/IPD (isolated postchallenge diabetes) |
| DHEA-Sd | DOWN | Serum | Chinese | 16 | GC/LC-MS/EISA | T2D/IPD (isolated postchallenge diabetes) |
| LysoPE(20:1/0:0)d | DOWN | Serum | Chinese | 16 | GC/LC-MS/EISA | T2D/IPD (isolated postchallenge diabetes) |
| LysoPE(20:2/0:0)d | DOWN | Serum | Chinese | 16 | GC/LC-MS/EISA | T2D/IPD (isolated postchallenge diabetes) |
| LysoPE(20:0/0:0)d | DOWN | Serum | Chinese | 16 | GC/LC-MS/EISA | T2D/IPD (isolated postchallenge diabetes) |
| LysoPC(18:0)d | DOWN | Serum | Chinese | 16 | GC/LC-MS/EISA | T2D/IPD (isolated postchallenge diabetes) |
| 5-Hydroxykynureninee | DOWN | Serum | Chinese | 16 | GC/LC-MS/EISA | T2D/IPD (isolated postchallenge diabetes) |
| Itaconic acid | UP | Plasma | Qatar | 17 | LC-MS | T2D |
| Inosine | DOWN | Plasma | Qatar | 17 | LC-MS | T2D |
| Uric acid | DOWN | Plasma | Qatar | 17 | LC-MS | T2D |
| Leucine | UP | Plasma | Qatar | 17 | LC-MS | T2D |
| 3-Hydroxymethylglutaric acid | DOWN | Plasma | Qatar | 17 | LC-MS | T2D |
| Succinate | DOWN | Plasma | Qatar | 17 | LC-MS | T2D |
| Taurine | DOWN | Plasma | Qatar | 17 | LC-MS | T2D |
| PC(18:0/0:0) | UP | Plasma | Qatar | 17 | LC-MS | T2D |
| Sphingosine-1-phosphate | UP | Plasma | Qatar | 17 | LC-MS | T2D |
| PE (P-16:0/22:6) | DOWN | Plasma | Qatar | 17 | LC-MS | T2D |
| PG (18:0/18:1) | UP | Plasma | Qatar | 17 | LC-MS | T2D |
| 2-Ketobutyric acid | DOWN | Urine | Qatar | 17 | LC-MS | T2D |
| 2-Ketoglutaric acid | DOWN | Urine | Qatar | 17 | LC-MS | T2D |
| 1-Methylhistidine | DOWN | Urine | Qatar | 17 | LC-MS | T2D |
| N-Acetyl-D-phenylalanine | UP | Urine | Qatar | 17 | LC-MS | T2D |
| Kynurenic acid | DOWN | Urine | Qatar | 17 | LC-MS | T2D |
| Xanthurenic acid | DOWN | Urine | Qatar | 17 | LC-MS | T2D |
| Serotonin | UP | Urine | Qatar | 17 | LC-MS | T2D |
| Pyruvic acid | DOWN | Urine | Qatar | 17 | LC-MS | T2D |
| Citrate | UP | Urine | Norway | 18 | NMR | GDM |

Reference:

1. Identification of serum metabolites associated with risk of type 2 diabetes using a targeted metabolomic approach. Floegel A, Stefan N,et al. Diabetes. 2013 Feb;62(2):639-48.

2. Novel biomarkers for pre-diabetes identified by metabolomics. Wang-Sattler R, Yu Z, et al. Mol Syst Biol. 2012;8:615.

3. Gut microbiome-derived metabolites characterize a peculiar obese urinary metabotype. Calvani R, Miccheli A, et al. Int J Obes (Lond). 2010 Jun;34(6):1095-8.

4. Metabolomic profiling of fatty acid and amino acid metabolism in youth with obesity and type 2 diabetes: evidence for enhanced mitochondrial oxidation. Mihalik SJ, Michaliszyn SF, et al. Diabetes Care. 2012 Mar;35(3):605-11.

5. Metabolite profiles and the risk of developing diabetes. Wang TJ, Larson MG, et al. Nat Med. 2011 Apr;17(4):448-53.

6. Metabolic footprint of diabetes: a multiplatform metabolomics study in an epidemiological setting. Suhre K, Meisinger C, et al.

PLoS One. 2010 Nov 11;5(11):e13953.

7. Metabonomic variations in the drug-treated type 2 diabetes mellitus patients and healthy volunteers. Bao Y, Zhao T, Wang X, Qiu Y, Su M, Jia W, Jia W. J Proteome Res. 2009 Apr;8(4):1623-30.

8. The furan fatty acid metabolite CMPF is elevated in diabetes and induces β cell dysfunction. Prentice KJ, Luu L, Allister EM, et al. Cell Metab. 2014 Apr 1;19(4):653-66.

9. Metabolic fingerprint of Gestational Diabetes Mellitus. Dudzik D, Zorawski M, et al. J Proteomics. 2014 May 30;103:57-71. d

10. 2-Aminoadipic acid is a biomarker for diabetes risk. Wang TJ, Ngo D, et al. J Clin Invest. 2013 Oct 1;123(10):4309-17."

11. Metabolomics study of type 2 diabetes using ultra-performance LC-ESI/quadrupole-TOF high-definition MS coupled with pattern recognition methods. Zhang AH, Sun H, Yet al. J Physiol Biochem. 2014 Mar;70(1):117-28.

12. Biomarkers for type 2 diabetes and impaired fasting glucose using a nontargeted metabolomics approach. Menni C, Fauman E,et al. Diabetes. 2013 Dec;62(12):4270-6.

13. Metabolite profiles during oral glucose challenge. Ho JE, Larson MG, Vasan RS,et al. Diabetes. 2013 Aug;62(8):2689-98.

14. Plasma and urine metabolic fingerprinting of type 1 diabetic children. Balderas C, Rupérez FJ,et al. Electrophoresis. 2013 Oct;34(19):2882-90.

15. Metabolic signature shift in type 2 diabetes mellitus revealed by mass spectrometry-based metabolomics. Xu F, Tavintharan S, Sum CF, Woon K, Lim SC, Ong CN. J Clin Endocrinol Metab. 2013 Jun;98(6):E1060-5.

16. Fasting serum lipid and dehydroepiandrosterone sulfate as important metabolites for detecting isolated postchallenge diabetes: serum metabolomics via ultra-high-performance LC-MS. Liu L, Wang M, Yang X, et al. Clin Chem. 2013 Sep;59(9):1338-48.

17. Quantitative metabolomic and lipidomic profiling reveals aberrant amino acid metabolism in type 2 diabetes. Kaur P, Rizk N, et al. Mol Biosyst. 2013 Feb 2;9(2):307-17.

18. Metabolic changes in urine during and after pregnancy in a large, multiethnic population-based cohort study of gestational diabetes.

Sachse D, Sletner L, et al. PLoS One. 2012;7(12):e52399.
