# Supplementary material for: Drug Repositioning for Diabetes Based on 'Omics' Data Mining
Source: PLoS One. 2015 May 6;10(5):e0126082. doi: 10.1371/journal.pone.0126082 (PMC4422696; doi:10.1371/journal.pone.0126082)
Supplement: S5 Table — (DOCX) [file pone.0126082.s005.docx]

**S5 Table.** 5 targets and 27 drugs have been used for diabetes treatment or at the stage of clinical trials

| **Drug name** | **Current drug indication** | **Stage** | **Target** | **Action mode** |
| --- | --- | --- | --- | --- |
| **Yohimbine** | Type2 diabetes, Sexual dysfunction | Phase II | Alpha-2A adrenergic receptor | antagonist |
| **Lisofylline** | Type 1 Diabetes | Phase I | Lysophosphatidic acid transferase | inhibitor |
| **AMG 151** | Type 2 diabetes | Phase II complete | Glucokinase | activator |
| **AZD1656** | Type 2 Diabetes | Phase II | Glucokinase | activator |
| **GK1-399** | Type 2 diabetes | Phase I/II | Glucokinase | activator |
| **AZD6370** | Type 2 Diabetes | Phase I completed | Glucokinase | activator |
| **AZD5658** | Obesity, Diabetes | Phase I | Glucokinase | activator |
| **DS-7309** | Diabetes | Phase I | Glucokinase | activator |
| **PSN-101** | Diabetes Mellitus Type 1 and 2 | Phase I | Glucokinase | activator |
| **TAK-329** | Diabetes mellitus | Phase I | Glucokinase | activator |
| **TAK-329** | Type 1 diabetes | Phase I | Glucokinase | activator |
| **TTP355** | Type 2 diabetes | Phase I | Glucokinase | activator |
| **Pioglitazone** | Diabetes mellitus | Approved | PPARG | agonist |
| **Rosiglitazone** | Diabetes mellitus | Approved | PPARG | agonist |
| **Troglitazone** | Diabetes mellitus | Approved | PPARG | agonist |
| **Rosiglitazone & metformin** | Type 2 diabetes | Phase III completed | PPARG | agonist |
| **Rosiglitazone & simvastatin** | Type 2 diabetes | Phase III completed | PPARG | agonist |
| **INT131** | Type 2 diabetes | Phase II | PPARG | agonist |
| **DB 959** | Type 2 diabetes | Phase I | PPARG | agonist |
| **DS-6930** | Diabetes | Phase I | PPARG | agonist |
| **DSP-8658** | Type 2 diabetes | Phase I | PPARG | agonist |
| **LY2605541** | Diabetes | Phase III | Insulin | analog |
| **LY2963016** | Diabetes | Phase III completed | Insulin | analog |
| **MK-1293** | Diabetes | Phase III | Insulin | analog |
| **NN1250** | Diabetes | Approved | Insulin | analog |
| **NN5401** | Diabetes | Approved | Insulin | analog |
| **NN1218** | Diabetes | Phase III | Insulin | analog |
| **Toujeo** | Diabetes | Phase III | Insulin | analog |
