# Supplementary material for: Drug Repositioning for Diabetes Based on 'Omics' Data Mining
Source: PLoS One. 2015 May 6;10(5):e0126082. doi: 10.1371/journal.pone.0126082 (PMC4422696; doi:10.1371/journal.pone.0126082)
Supplement: S6 Table — (DOCX) [file pone.0126082.s006.docx]

**S6 Table**. 30 proteins corresponding to 167 drug projects might be repurposed for novel indications for treating diabetes

| **T2D associated genes/proteins** | **Disease** | **Drug name** | **Type** | **Target** | **Action mode** |
| --- | --- | --- | --- | --- | --- |
| ADRA2A | Attention deficit hyperactivity disorder | Connexyn | Approved | Alpha-2A adrenergic receptor | agonist |
|  | anti-Parkinsonian | Atipamezole | research | Alpha-2A adrenergic receptor | antagonist |
|  | hypertension,hypoplastic left heart syndrome. | Phenoxybenzamine | Aproved | Alpha-2A adrenergic receptor | antagonist |
|  | Major Depressive Disorder | Idazoxan | Phase III withdraw | Alpha-2A adrenergic receptor | antagonist |
| IGF2BP2 | Fibrosis | EXC 001 | Phase II | mRNA of IGFBP-8 | Antisense |
| KCNQ1 | Hypertension | Indapamide | Approved | Potassium voltage-gated channel subfamily KQT member 1 | Blocker |
|  | Multiple Sclerosis | Nerispirdine | Phase II | Potassium voltage-gated channel subfamily KQT member 1 | Blocker |
| PLA2G1B | Inflammation and itching | Clobetasol | Approved | Phospholipase A2 | inhibitor |
|  | Inflammatory skin conditions | Clocortolone | Approved | Phospholipase A2 | inducer |
|  | Atopic dermatitis | Desonide | Approved | Phospholipase A2 | inhibitor |
|  | Inflammatory diseases | Desoximetasone | Approved | Phospholipase A2 | inhibitor |
|  | Skin Allergies | Diflorasone | Approved | Phospholipase A2 | inhibitor |
|  | Inflammatory diseases | Halobetasol Propionate | Approved | Phospholipase A2 | inhibitor |
|  | Inflammatory diseases | Hydrocortamate | Approved | Phospholipase A2 | inhibitor |
|  | Inflammatory diseases | Prednicarbate | Approved | Phospholipase A2 | inducer |
|  | Giardiasis and cutaneous leishmaniasis | Quinacrine | Approved | Phospholipase A2 | inhibitor |
|  | Visceral Leishmaniasis, Fungal diseases | Miltefosine | Phase II | Phospholipase A2 | inhibitor |
|  | Coronary Artery Disease, Atherosclerosis | Varespladib | Phase II | Phospholipase A2,membrane associated | inhibitor |
| BCHE | Chronic glaucoma | Echothiophate Iodide | Approved | Cholinesterase | inhibitor |
|  | Spasms, Pain | Hexafluronium bromide | Approved | Cholinesterase | inhibitor |
| NOS1 | Migraine and Cluster Headaches | NXN-188 | Phase I | Nitric oxide synthase, brain | Inhibitor |
|  | Angina, Coronary Artery Disease | ACCLAIM | Phase III | Nitric-oxide synthase, endothelial | Stimulator |
|  | Angina | L-NMMA | Phase III | Nitric-oxide synthase, endothelial | inhibitor |
|  | Hypertension, Angina | L-NAME | Phase II/III | Nitric-oxide synthase, endothelial | inhibitor |
|  | Inflammatory diseases | Hydrocortisone | Approved | Nitric oxide synthase, inducible | inhibitor |
| PTGS2 | Pain | carprofen | Approved | Prostaglandin G/H synthase 2 | inhibitor |
|  | Rheumatoid arthritis and osteoarthritis | Celecoxib | Approved | Prostaglandin G/H synthase 2 | inhibitor |
|  | Pain | Diflunisal | Approved | Prostaglandin G/H synthase 2 | inhibitor |
|  | Pain | Etodolac | Approved | Prostaglandin G/H synthase 2 | inhibitor |
|  | Rheumatoid arthritis and osteoarthritis | Etoricoxib | Approved | Prostaglandin G/H synthase 2 | inhibitor |
|  | Pain | Ibuprofen | Approved | Prostaglandin G/H synthase 2 | inhibitor |
|  | Rheumatoid arthritis and pain | Ketoprofen | Approved | Prostaglandin G/H synthase 2 | inhibitor |
|  | Knee osteoarthritis | Lumiracoxib | Approved | Prostaglandin G/H synthase 2 | inhibitor |
|  | Rheumatoid arthritis and osteoarthritis | Mefenamic acid | Approved | Prostaglandin G/H synthase 2 | inhibitor |
|  | Arthritis | Meloxicam | Approved | Prostaglandin G/H synthase 2 | inhibitor |
|  | Rheumatoid arthritis and osteoarthritis | Nabumetone | Approved | Prostaglandin G/H synthase 2 | inhibitor |
|  | Pain and Rheumatoid arthritis | Naproxen | Approved | Prostaglandin G/H synthase 2 | inhibitor |
|  | Rheumatoid arthritis | Niflumic Acid | Approved | Prostaglandin G/H synthase 2 | inhibitor |
|  | Chronic pain | Phenylbutazone | Approved | Prostaglandin G/H synthase 2 | inhibitor |
|  | Pain | Piroxicam | Approved | Prostaglandin G/H synthase 2 | inhibitor |
|  | Rheumatoid arthritis and osteoarthritis | Tenoxicam | Approved | Prostaglandin G/H synthase 2 | inhibitor |
|  | Pain | Tiaprofenic acid | Approved | Prostaglandin G/H synthase 2 | inhibitor |
|  | Rheumatoid arthritis and osteoarthritis | Tolmetin | Approved | Prostaglandin G/H synthase 2 | inhibitor |
|  | Osteoarthritis and rheumatoid arthritis | Valdecoxib | Approved | Prostaglandin G/H synthase 2 | inhibitor |
|  | Stroke | ONO-2506 | Phase III completed | Prostaglandin G/H synthase 2 | inhibitor |
|  | Pain | Celecoxib | Phase III | Prostaglandin G/H synthase 2 | inhibitor |
|  | Neuropathic pain | GSK-644784 | Suspended in Phase II in GSK 2006 Report | Prostaglandin G/H synthase 2 | inhibitor |
|  | Osteoarthritis, Neuropathic pain | GW-406381 | Suspended in Phase III in GSK 2006 Report | Prostaglandin G/H synthase 2 | inhibitor |
|  | Osteoarthritis | Rofecoxib | Withdrawn | Prostaglandin G/H synthase 2 | inhibitor |
|  | Pain | C-myb | Experimental | Prostaglandin G/H synthase 2 | upregulator |
| PAFAH1B2 | Atherosclerosis | Darapladib | Phase III | Platelet-activating factor acetylhydrolase | inhibitor |
|  | Atherosclerosis | Rilapladib | Phase II | Platelet-activating factor acetylhydrolase | inhibitor |
|  | Cardiovascular Disorders | GSK 659032 | Phase I | Platelet-activating factor acetylhydrolase | inhibitor |
| GRIN2A | Dietary shortage | Glycine | Approved | Glutamate [NMDA] receptor subunit epsilon 1 (NMDAR2A) | agonist |
|  | Attention deficit hyperactivity disorder | Amphetamine | Approved | NMDA receptor | Antagonist |
|  | Bacterial infections | D-cycloserine | Approved | NMDA receptor | agonist |
|  | Epilepsy | Felbamate | Approved | NMDA receptor | Antagonist |
|  | Anesthetic | Ketamine | Approved | NMDA receptor | Antagonist |
|  | Schizophrenia | L-Glutamic Acid | Approved | NMDA receptor | Antagonist |
|  | Acute pain | Magnesium Sulfate | Approved | NMDA receptor | Antagonist |
|  | Alzheimer's disease | Memantine | Approved | NMDA receptor | Antagonist |
|  | Anaesthesia | Nitrous oxide | Approved | NMDA receptor | Antagonist |
|  | Parkinson's disease | Orphenadrine | Approved | NMDA receptor | Antagonist |
|  | Painful diabetic neuropathy | Dextromethorphan+quinidine | Phase III completed | NMDA receptor | Antagonist |
|  | Neuropathic pain | Amitriptyline+ketamine | Phase III | NMDA receptor | Antagonist |
|  | Traumatic brain injury | Dexanabinol | Phase III | NMDA receptor | Antagonist |
|  | Acute ischemic stroke | Magnesium | Phase III | NMDA receptor | Antagonist |
|  | Neuropathic, Pain | Memantine | Phase III | NMDA receptor | Antagonist |
|  | Major Depressive Disorder | AZD6765 | Phase II | NMDA receptor | Antagonist |
|  | Neuropathic pain | CHF-3381 | Phase II | NMDA receptor | Antagonist |
|  | Neuropathic pain | CNS-5161 | Phase II | NMDA receptor | Antagonist |
|  | Obsessive-compulsive disorder | D-cycloserine | Phase II | NMDA receptor | agonist |
|  | Schizophrenia | D-serine | Phase II | NMDA receptor | agonist |
|  | Cancer pain | Neramexane | Phase II | NMDA receptor | Antagonist |
|  | Painful diabetic neuropathy | EAA-090 | Terminated in Phase II | NMDA receptor | Antagonist |
|  | Parkinson's Disease | Remacemide | Discontinued in Phase I | NMDA receptor | agonist |
|  | Huntington¡¯s disease | Remacemide | Discontinued in Phase III | NMDA receptor | agonist |
| MPO | Parkinson's Disease | AZD3241 | Phase I | Myeloperoxidase | inhibitor |
|  | Multiple Sclerosis | AZD5904 | Phase I discontinue | Myeloperoxidase | inhibitor |
| CD1a | Aplastic anemia | Antithymocyte globulin | Approved | T-cell surface glycoprotein CD1a | binder |
| 5-HT-1A | Anxiety disorder | Buspirone | Approved | Serotonin-1A | agonist |
|  | Migraine | Treximet | Approved | Serotonin-1A | antagonist |
|  | Major depressive disorder | Vilazodone | Phase III | Serotonin-1A | antagonist |
|  | Alzheimer Disease,Peripheral sensory | Xaliproden | Phase III | Serotonin-1A | antagonist |
|  | Female sexual dysfunction | Flibanserin | Phase III | Serotonin-1A | agonist |
|  | Major depressive disorder $ major depressive episode $ anxiety | GSK163090 | Phase II | Serotonin-1A | antagonist |
|  | Severe Mood disorder | MN-305 | Phase II | Serotonin-1A | agonist |
|  | Bulimia nervosa OCD MDD, severe mood disorders | OPC-14523 | Phase II | Serotonin-1A | agonist |
|  | Severe Mood disorder | TGBA01AD | Phase II | Serotonin-1A | agonist |
|  | Female sexual dysfunction | OPC-14523 | Phase I | Serotonin-1A | agonist |
|  | Schizophrenia | SSR-181507 | Phase I | Serotonin-1A | binder |
|  | Schizophrenia | 1192U90 | Discontinued | Serotonin-1A | agonist |
|  | Severe Mood disorder | Adatanserin | Discontinued in phase II | Serotonin-1A | agonist |
|  | Schizophrenia | Bifeprunox | Teminated in phase III | Serotonin-1A | agonist |
|  | Severe Mood disorder | PRX-00023 | Discontinued in phase II | Serotonin-1A | agonist |
|  | Schizophrenia | SLV-313 | Terminated in phase I | Serotonin-1A | agonist |
|  | Obesity, Psychosis | SLV-319 | Suspended in phase I | Serotonin-1A | Antagonist |
|  | Parkinson's Disease | Sarizotan | Discontinued in phase II | Serotonin-1A | agonist |
|  | Cognition impairment associated | AV965 | Phase I | Serotonin-1A | ? |
| MAO-A | Parkinson's disease | Clorgyline | Approved | Amine oxidase [flavin-containing] A | inhibitor |
|  | Depression | Isocarboxazid | Approved | Amine oxidase [flavin-containing] A | inhibitor |
|  | Depression | Moclobemide | Approved | Amine oxidase [flavin-containing] A | inhibitor |
|  | Depression | Tranylcypromine | Approved | Amine oxidase [flavin-containing] A | inhibitor |
|  | Neuropathic pain | CHF-3381 | Phase II | Amine oxidase [flavin-containing] A | inhibitor |
| ABCB4 | Acute Myeloid Leukemia | LY335979 | Phase III | P-glycoprotein | inhibitor |
| 5HTT | Major Depressive Disorder | Vilazodone | Phase III | Sodium-dependent serotonin transporter | inhibitor |
|  | Depression | LY367265 | Discontinued | Sodium-dependent serotonin transporter | inhibitor |
|  | Schizophrenia | SLV-310 | Suspended in phase II | Sodium-dependent serotonin transporter | inhibitor |
|  | Depression | YM-992 | Discontinued in phase II | Sodium-dependent serotonin transporter | inhibitor |
| 5HT 2B | Depression | Minaprine | Approved | 5-hydroxytryptamine 2B receptor | Antagonist |
|  | Psychosis | Triflupromazine | Approved | 5-hydroxytryptamine 2B receptor | Antagonist |
|  | Pulmonary arterial hypertension | PRX-08066 | Phase II | 5-hydroxytryptamine 2B receptor | Antagonist |
| DHOD | Malaria | Artemisinin | Approved | Dihydroorotate dehydrogenase, mitochondrial | inhibitor |
|  | Fungal infections | Atovaquone | Approved | Dihydroorotate dehydrogenase, mitochondrial | inhibitor |
|  | Active rheumatoid arthritis | Leflunomide | Approved | Dihydroorotate dehydrogenase, mitochondrial | inhibitor |
|  | Rheumatoid arthritis | Teriflunomide | Phase III | Dihydroorotate dehydrogenase, mitochondrial | inhibitor |
|  | Rheumatoid arthritis, inflammatory bowel disease | Vidofludimus | Phase II | Dihydroorotate dehydrogenase, mitochondrial | inhibitor |
|  | Advanced gastrointestinal cancer | Brequinar sodium | Discontinued in phase II | Dihydroorotate dehydrogenase, mitochondrial | inhibitor |
|  | Kidney Transplantation, Heart Transplantation | FK778 | Terminated in phase II | Dihydroorotate dehydrogenase, mitochondrial | inhibitor |
| 5-HT-4 | Gastroesophageal | Cisapride | Approved | 5-hydroxytryptamine 4 receptor | agonist |
|  | Irritable bowel syndrome | Tegaserod | Approved | 5-hydroxytryptamine 4 receptor | agonist |
|  | Irritable bowel syndrome | Renzapride | Phase III | 5-hydroxytryptamine 4 receptor | Antagonist |
|  | Dyspepsia | Tegaserod | Phase III | 5-hydroxytryptamine 4 receptor | agonist |
|  | Psoriasis | Fluphenazine Decanoate | Phase II | 5-hydroxytryptamine 4 receptor | Antagonist |
|  | Alzheimer's disease | PRX-3140 | Phase II | 5-hydroxytryptamine 4 receptor | ? |
|  | Irritable bowel syndrome | Pumosetrag | Phase II | 5-hydroxytryptamine 4 receptor | agonist |
|  | Gastro-oesophagal reflux | Tegaserod | Phase II | 5-hydroxytryptamine 4 receptor | agonist |
|  | Gastro-oesophagal reflux | Pumosetrag | Phase I | 5-hydroxytryptamine 4 receptor | agonist |
|  | Cognitive impairment associated with Alzheimer's disease | TD-8954 | Phase I | 5-hydroxytryptamine 4 receptor | ? |
| IL2RB | Cancer/Tumors | Medusa IL-2 | Phase I/II | Interleukin-2 receptor subunit beta | agonist |
| CTLA4 | Late-stage melanoma | Ipilimumab | Approved | Cytotoxic T-lymphocyte protein 4 | antibody |
|  | Genitourinary, Gastrointestinal Cancers, Colorectal Cancer, Melanoma and Cell Carcinoma | CP-675206 | Phase II | Cytotoxic T-lymphocyte protein 4 | antibody |
|  | Solid Tumors | Tremelimumab | Phase II | Cytotoxic T-lymphocyte protein 4 | ? |
| TYK2 | Solid Tumors | BI-853520 | Phase I | TYK2 | ? |
| CCR5 | HIV infection | Maraviroc | Approved | C-C chemokine receptor type 5 | Antagonist |
|  | Chronic obstructive pulmonary disease | Maraviroc | Phase III | C-C chemokine receptor type 5 | Antagonist |
|  | HIV Infection | Vicriviroc | Phase III | C-C chemokine receptor type 5 | Antagonist |
|  | Infections | INCB9471 | Phase II completed | C-C chemokine receptor type 5 | Antagonist |
|  | HIV Infection | PRO 140 | Phase II completed | C-C chemokine receptor type 5 | antibody |
|  | HIV-1 infection treatment | Cenicriviroc | Phase II | C-C chemokine receptor type 5 | ? |
|  | HIV infection treatment | RAP101 | Phase II | C-C chemokine receptor type 5 | ? |
|  | HIV Infection | SCH-D | Phase II | C-C chemokine receptor type 5 | Antagonist |
|  | Chronic obstructive pulmonary disease; Rheumatoid arthritis | AZD8566 | Phase I completed | C-C chemokine receptor type 5 | Antagonist |
|  | HIV-1 | Tak-220 | Phase I | C-C chemokine receptor type 5 | binder |
|  | Rheumatoid arthritis | AZD5672 | Discontinued in Phase I | C-C chemokine receptor type 5 | Antagonist |
|  | HIV-1 Infection | GW873140 | Terminated in Phase III | C-C chemokine receptor type 5 | binder |
|  | HIV infection | INCB15050 | Discontinued in Phase I | C-C chemokine receptor type 5 | Antagonist |
|  | HIV Infection | SCH-C | Discontinued | C-C chemokine receptor type 5 | binder |
|  | HIV Infection | Sch-351125 | Discontinued | C-C chemokine receptor type 5 | Antagonist |
|  | HIV Infection | TAK-779 | Discontinued | C-C chemokine receptor type 5 | Antagonist |
| PRKCQ | Renal Transplant | Sotrastaurin acetate | Phase II | Protein kinase C, theta type | inhibitor |
| SERPINC1 | Acute coronary syndrome | Heparin | Approved | Antithrombin-III | cofactor |
|  | Central or sensorineural tinnitus | Sulodexide | Approved | Antithrombin-III | activator |
|  | Deep vein thrombosis and pulmonary embolism | Tinzaparin | Approved | Antithrombin-III | cofactor |
| SERPINA1 | Emphysema | Alpha1-proteinase inhibitor | Approved | Alpha-1-antitrypsin | inhibitor |
|  | Emphysema | Zemaira | Approved | Alpha-1-antitrypsin | inhibitor |
|  | Chronic obstructive pulmonary disease, cystic fibrosis | Alphagen | Phase I | Alpha-1-antitrypsin | Modulator |
| HSPB1 | Breast, ovarian, bladder, prostate or lung cancer | OGX-427 | Phase II | mRNA of Heat shock 27 kDa protein | Antisense |
| INDO | Depression | L-Tryptophan | Approved | Indoleamine 2,3-dioxygenase | binder |
|  | Oncology | INCB24360 | Phase I | Indoleamine 2,3-dioxygenase | inhibitor |
| CPT1A | Nutritional supplement | Approved | L-Carnitine | Carnitine O-palmitoyltransferase I | activator |
|  | Angina pectoris | Approved | Perhexiline | Carnitine O-palmitoyltransferase I | inhibitor |
| PLA2G7 | Atherosclerosis | arapladib | Phase III | Platelet-activating factor acetylhydrolase | inhibitor |
|  | Atherosclerosis | Rilapladib | Phase II | Platelet-activating factor acetylhydrolase | inhibitor |
|  | Cardiovascular Disorders | GSK 659032 | Phase I | Platelet-activating factor acetylhydrolase | inhibitor |
|  | Atherosclerosis | GSK568859 | Phase I | Platelet-activating factor acetylhydrolase | inhibitor |
| MTNR1B | Insomnia | Ramelteon | Approved | Melatonin receptor type 1B | agonist |
