# Supplementary material for: Drug Repositioning for Diabetes Based on 'Omics' Data Mining
Source: PLoS One. 2015 May 6;10(5):e0126082. doi: 10.1371/journal.pone.0126082 (PMC4422696; doi:10.1371/journal.pone.0126082)
Supplement: S7 Table — (DOCX) [file pone.0126082.s007.docx]

**S7 Table.** CMap analysis of 58 drugs.

| **Drug name** | **Drug target** | **Omics method** | **Current drug indication** | **cmAP** | **P-Value** |
| --- | --- | --- | --- | --- | --- |
| **Phenoxybenzamine** | Alpha-2A adrenergic receptor | GWAS | hypertension, hypoplastic left heart syndrome | resveratrol (0.799) | 0.034 |
| **Idazoxan** | Alpha-2A adrenergic receptor | GWAS | Major Depressive Disorder | gliclazide (0.728) | 0.011 |
| **Clobetasol** | Phospholipase A2 | Metabolomics | Inflammation and itching | ns |  |
| **Desonide** | Phospholipase A2 | Metabolomics | Atopic dermatitis | na |  |
| **Desoximetasone** | Phospholipase A2 | Metabolomics | Inflammatory diseases | na |  |
| **Diflorasone** | Phospholipase A2 | Metabolomics | Skin Allergies | streptozocin (-0.709) | 0.015 |
| **Halobetasol Propionate** | Phospholipase A2 | Metabolomics | Inflammatory diseases | na |  |
| **Hydrocortamate** | Phospholipase A2 | Metabolomics | Inflammatory diseases | na |  |
| **Quinacrine** | Phospholipase A2 | Metabolomics | Giardiasis and cutaneous leishmaniasis | na |  |
| **Miltefosine** | Phospholipase A2 | Metabolomics | Visceral Leishmaniasis, Fungal diseases | na |  |
| **Varespladib** | Phospholipase A2 | Metabolomics | Coronary Artery Disease, Atherosclerosis | na |  |
| **Echothiophate Iodide** | Cholinesterase | Metabolomics | Chronic glaucoma | na |  |
| **Hexafluronium bromide** | Cholinesterase | Metabolomics | Spasms, Pain | na |  |
| **Hydrocortisone** | Nitric oxide synthase, inducible | Metabolomics | Inflammatory diseases | na |  |
| **carprofen** | Prostaglandin G/H synthase 2 | Metabolomics | Pain | na |  |
| **Celecoxib** | Prostaglandin G/H synthase 2 | Metabolomics | Rheumatoid arthritis and osteoarthritis | ns |  |
| **Diflunisal** | Prostaglandin G/H synthase 2 | Metabolomics | Pain | glimepiride (0.626) | 0.049 |
| **Etodolac** | Prostaglandin G/H synthase 2 | Metabolomics | Pain | ns |  |
| **Etoricoxib** | Prostaglandin G/H synthase 2 | Metabolomics | Rheumatoid arthritis and osteoarthritis | na |  |
| **Ibuprofen** | Prostaglandin G/H synthase 2 | Metabolomics | Pain | na |  |
| **Ketoprofen** | Prostaglandin G/H synthase 2 | Metabolomics | Rheumatoid arthritis and pain | na |  |
| **Lumiracoxib** | Prostaglandin G/H synthase 2 | Metabolomics | Knee osteoarthritis | na |  |
| **Mefenamic acid** | Prostaglandin G/H synthase 2 | Metabolomics | Rheumatoid arthritis and osteoarthritis | ns |  |
| **Meloxicam** | Prostaglandin G/H synthase 2 | Metabolomics | Arthritis | na |  |
| **Nabumetone** | Prostaglandin G/H synthase 2 | Metabolomics | Rheumatoid arthritis and osteoarthritis | resveratrol (0.576) | 0.002 |
| **Naproxen** | Prostaglandin G/H synthase 2 | Metabolomics | Pain and Rheumatoid arthritis | ns |  |
| **Niflumic Acid** | Prostaglandin G/H synthase 2 | Metabolomics | Rheumatoid arthritis | resveratrol (0.484) | 0.018 |
| **Phenylbutazone** | Prostaglandin G/H synthase 2 | Metabolomics | Chronic pain | na |  |
| **Piroxicam** | Prostaglandin G/H synthase 2 | Metabolomics | Pain | ns |  |
| **Tenoxicam** | Prostaglandin G/H synthase 2 | Metabolomics | Rheumatoid arthritis and osteoarthritis | ns |  |
| **Tiaprofenic acid** | Prostaglandin G/H synthase 2 | Metabolomics | Pain | ns |  |
| **Tolmetin** | Prostaglandin G/H synthase 2 | Metabolomics | Rheumatoid arthritis and osteoarthritis | ns |  |
| **Valdecoxib** | Prostaglandin G/H synthase 2 | Metabolomics | Osteoarthritis and rheumatoid arthritis | metformin (0.412) | 0.047 |
| **ONO-2506** | Prostaglandin G/H synthase 2 | Metabolomics | Stroke | na |  |
| **Celecoxib** | Prostaglandin G/H synthase 2 | Metabolomics | Pain | ns |  |
| **GSK-644784** | Prostaglandin G/H synthase 2 | Metabolomics | Neuropathic pain | na |  |
| **GW-406381** | Prostaglandin G/H synthase 2 | Metabolomics | Osteoarthritis, Neuropathic pain | na |  |
| **Rofecoxib** | Prostaglandin G/H synthase 2 | Metabolomics | Osteoarthritis | ns |  |
| **D-cycloserine** | NMDA receptor | Metabolomics | Bacterial infections | gliclazide (0.65) | 0.036 |
| **D-cycloserine** | NMDA receptor | Metabolomics | Obsessive-compulsive disorder | gliclazide (0.65) | 0.036 |
| **D-serine** | NMDA receptor | Metabolomics | Schizophrenia | na |  |
| **Remacemide** | NMDA receptor | Metabolomics | Parkinson's Disease | na |  |
| **Remacemide** | NMDA receptor | Metabolomics | Huntington's disease | na |  |
| **Buspirone** | Serotonin-1A | Metabolomics | Anxiety disorder | ns |  |
| **Flibanserin** | Serotonin-1A | Metabolomics | Female sexual dysfunction | na |  |
| **MN-305** | Serotonin-1A | Metabolomics | Severe Mood disorder | na |  |
| **OPC-14523** | Serotonin-1A | Metabolomics | Bulimia nervosa OCD MDD, severe mood disorders | na |  |
| **TGBA01AD** | Serotonin-1A | Metabolomics | Severe Mood disorder | na |  |
| **OPC-14523** | Serotonin-1A | Metabolomics | Female sexual dysfunction | na |  |
| **1192U90** | Serotonin-1A | Metabolomics | Schizophrenia | na |  |
| **Adatanserin** | Serotonin-1A | Metabolomics | Severe Mood disorder | na |  |
| **Bifeprunox** | Serotonin-1A | Metabolomics | Schizophrenia | na |  |
| **PRX-00023** | Serotonin-1A | Metabolomics | Severe Mood disorder | na |  |
| **SLV-313** | Serotonin-1A | Metabolomics | Schizophrenia | na |  |
| **Sarizotan** | Serotonin-1A | Metabolomics | Parkinson's Disease | na |  |
| **Perhexiline** | Carnitine O-palmitoyltransferase I | Metabolomics | Angina pectoris | resveratrol(0.697) | 0.00006 |
| **Cisapride** | 5-hydroxytryptamine 4 receptor | Metabolomics | Gastroesophageal | ns |  |
| **Medusa IL-2** | Interleukin-2 receptor subunit beta | Metabolomics | Cancer/Tumors | na |  |
| **Sotrastaurin acetate** | Protein kinase C, theta type | Metabolomics | Renal Transplant | na |  |
| **Ramelteon** | Melatonin receptor type 1B**^*^** | GWAS | Insomnia | na |  |

* This protein was repurposed for diabetes treatment before.
